# Supplementary figures and images for: Nuclear elongation during spermiogenesis depends on physical linkage of nuclear pore complexes to bundled microtubules by Drosophila Mst27D
Source: PLoS Genet. 2023 Jul 10;19(7):e1010837. doi: 10.1371/journal.pgen.1010837 (PMC10359004; doi:10.1371/journal.pgen.1010837)

# mCherry-Nup358

FL

N1

N4

N5

C1

C2

C4

C5

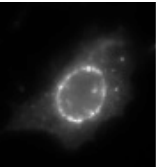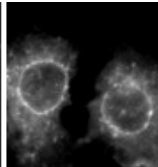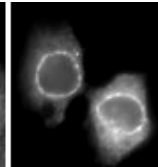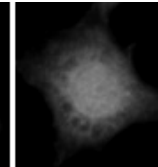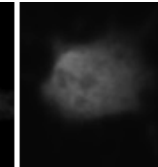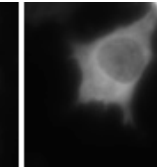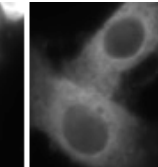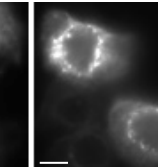

Supplement: S1 Fig — S2R+ cells transiently expressing the indicated Nup358 fragments tagged with mCherry at the N terminus (see Fig 2B for additional information) were fixed for microscopic analysis. While expression levels varied substantially from cell to cell, the displayed images are from cells with relatively weak mCherry signals, in which NE enrichment was most clearly detectable if present. Fragments N1 –N4 were strongly enriched at the NE, comparable to full length mCherry-Nup358. In contrast, N5 and C1 –C4 were at most very weakly enriched at the NE. C5, which includes the OE but not the NTD, was usually in sheet-like aggregates that were perinuclear in cells with less extensive aggregates. Transient expression of C1 –C5 was also accompanied by an accumulation of apoptotic S2R+ cells that was not observed with N1 –N5. Scale bar = 5 μm. (PDF) [file pgen.1010837.s001.pdf]

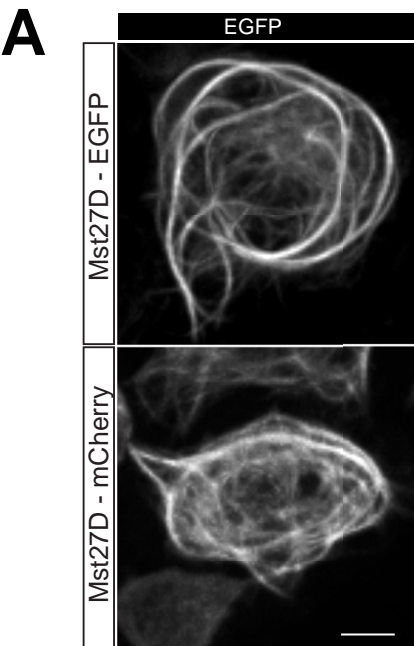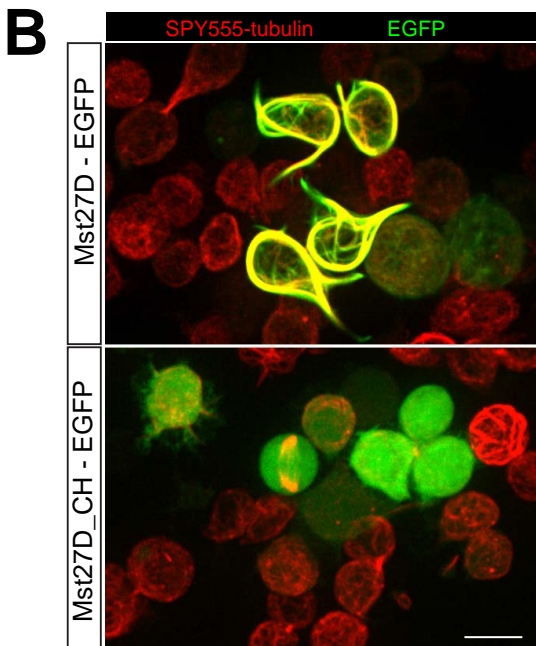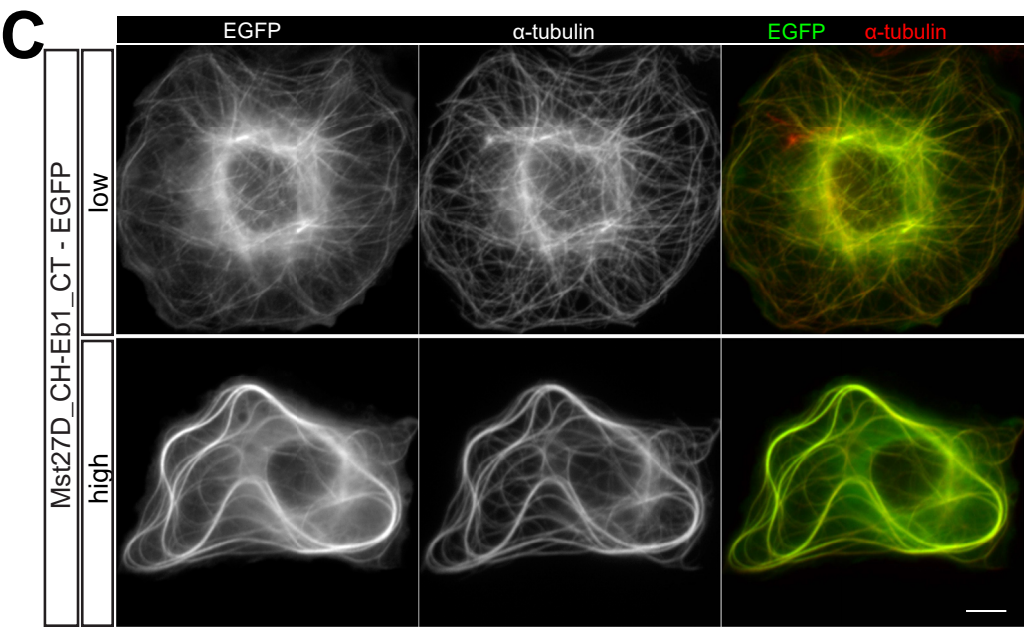

Supplement: S2 Fig — (A) S2R+ cell lines expressing either Mst27D-EGFP or Mst27D-mCherry were analyzed by live imaging. Still frames display cells with prominent intracellular cables that were observed in cells characterized by high expression levels. Scale bar = 5 μm. (B) S2R+ cell lines expressing either Mst27D-EGFP or Mst27D_CH-EGFP were incubated with the MT live stain SPY555-tubulin before live imaging. Cells with high levels of Mst27D-EGFP display prominent MT cables in contrast to cells with high levels of Mst27D_CH-EGFP. Scale bar = 10 μm. (C) S2R+ cell lines expressing Mst27D_CH-Eb1_CT-EGFP, the EGFP tagged chimeric protein with the CH domain of Mst27D and the CT region of D. melanogaster Eb1, were fixed and double labeled with anti-α-tubulin. A weakly (top) and a strongly (bottom) expressing ceIl are shown, revealing the bundling of MTs into prominent intracellular cables at high expression levels. Scale bar = 5 μm. (PDF) [file pgen.1010837.s002.pdf]

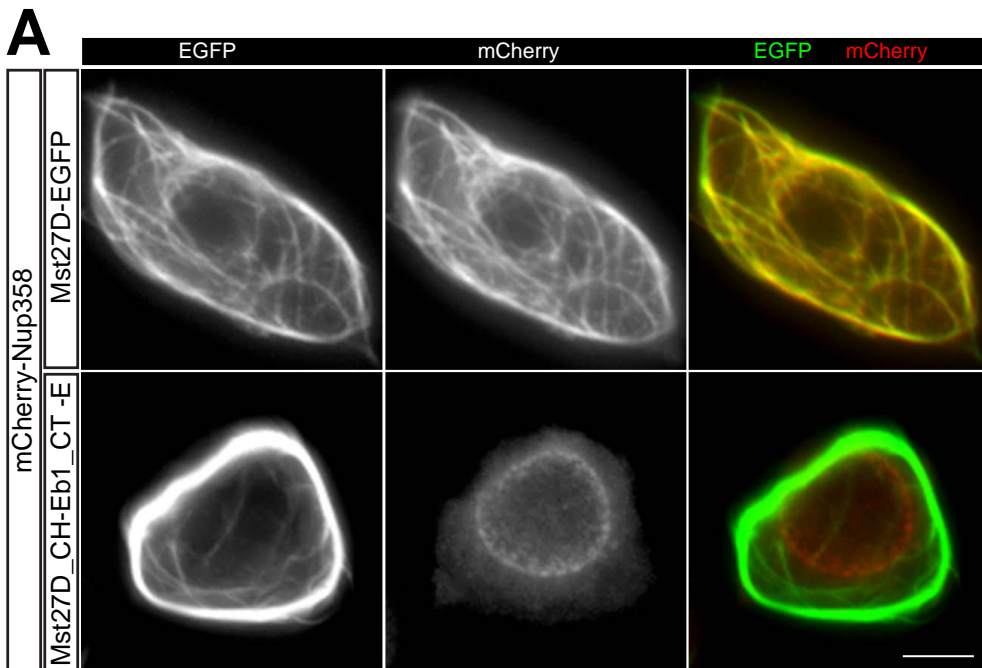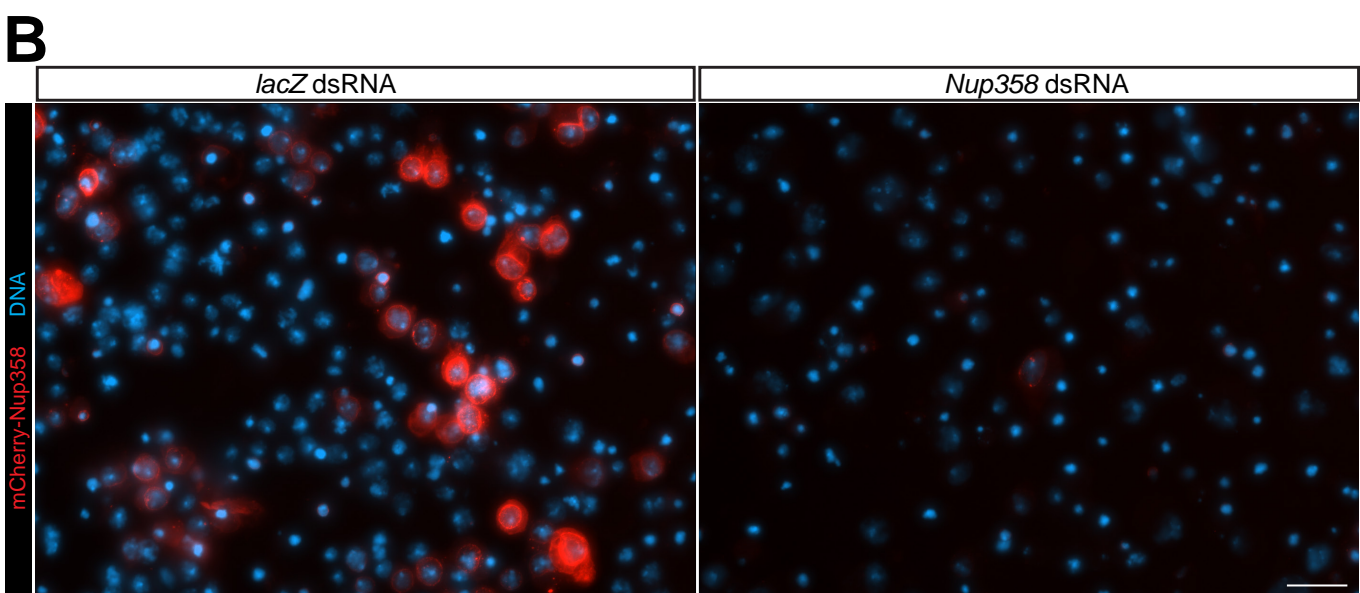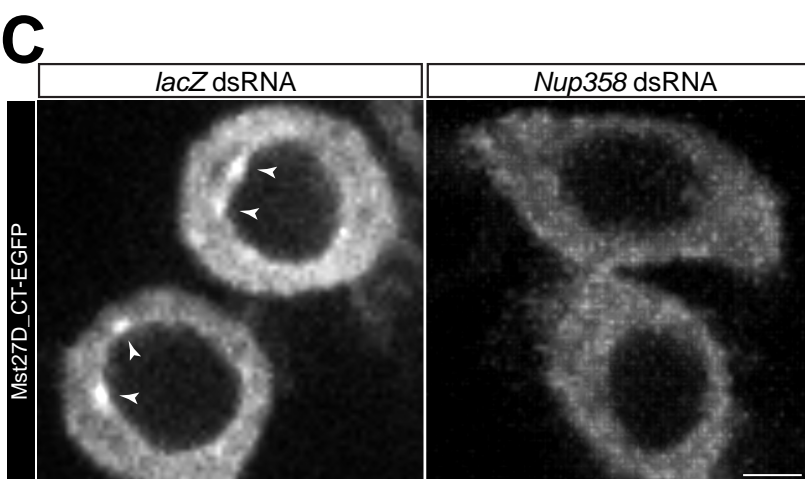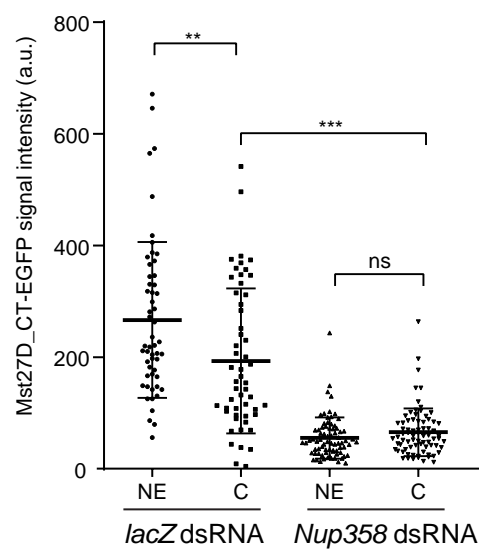

Supplement: S3 Fig — (A) S2R+ cells transiently co-expressing mCherry-Nup358 and either Mst27D-EGFP or Mst27D_CH-Eb1_CT-EGFP were fixed for microscopic analysis. Representative cells with strong MT cables are displayed. While mCherry-Nup358 is recruited to the MT cables induced by Mst27D-EGFP at high levels of expression, mCherry-Nup358 is not recruited to MT cables induced by high levels of Mst27D_CH-Eb1_CT-EGFP expression, as expected because the chimeric protein lacks the CT region of Mst27D that mediates binding to Nup358. (B,C) Effects of Nup358 depletion by RNAi in S2R+ cells. (B) Incubation of S2R+ cells with Nup358 dsRNA results in the expected depletion of Nup358. S2R+ cells stably expressing mCherry-Nup358 were treated with either Nup358 or lacZ dsRNA for control. DNA was labeled after fixation. (C) Nup358 depletion abolishes the enrichment of Mst27D_CT-EGFP at the NE. S2R+ cells stably expressing Mst27D_CT-EGFP were treated with either Nup358 or lacZ dsRNA before live imaging of EGFP signals. Single optical sections through representative cells are displayed. The weak enrichment of Mst27D_CT-EGFP on the nuclear rim and more prominently on putative annulate lamellae (arrowheads) that is evident in lacZ dsRNA treated controls is absent after treatment with Nup358 dsRNA. EGFP signal intensities associated with the NE and within the cytoplasm were quantified. Swarm plots display values from individual cells; mean and s.d. are indicated as well. n = 54 (lacZ dsRNA) and 76 (Nup358 dsRNA). Nup358 depletion also reduced the overall level of Mst27D_CT-EGFP, suggesting that Nup358 is required for normal expression of Mst27D_CT-EGFP, perhaps reflecting a Nup358 requirement for normal translation, as recently shown with human HCT116 cells [37]. Scale bars = 5 μm (A), 20 μm (B) and 3 μm (C). (PDF) [file pgen.1010837.s003.pdf]

# **A** *g-Mst27D-Dendra2*

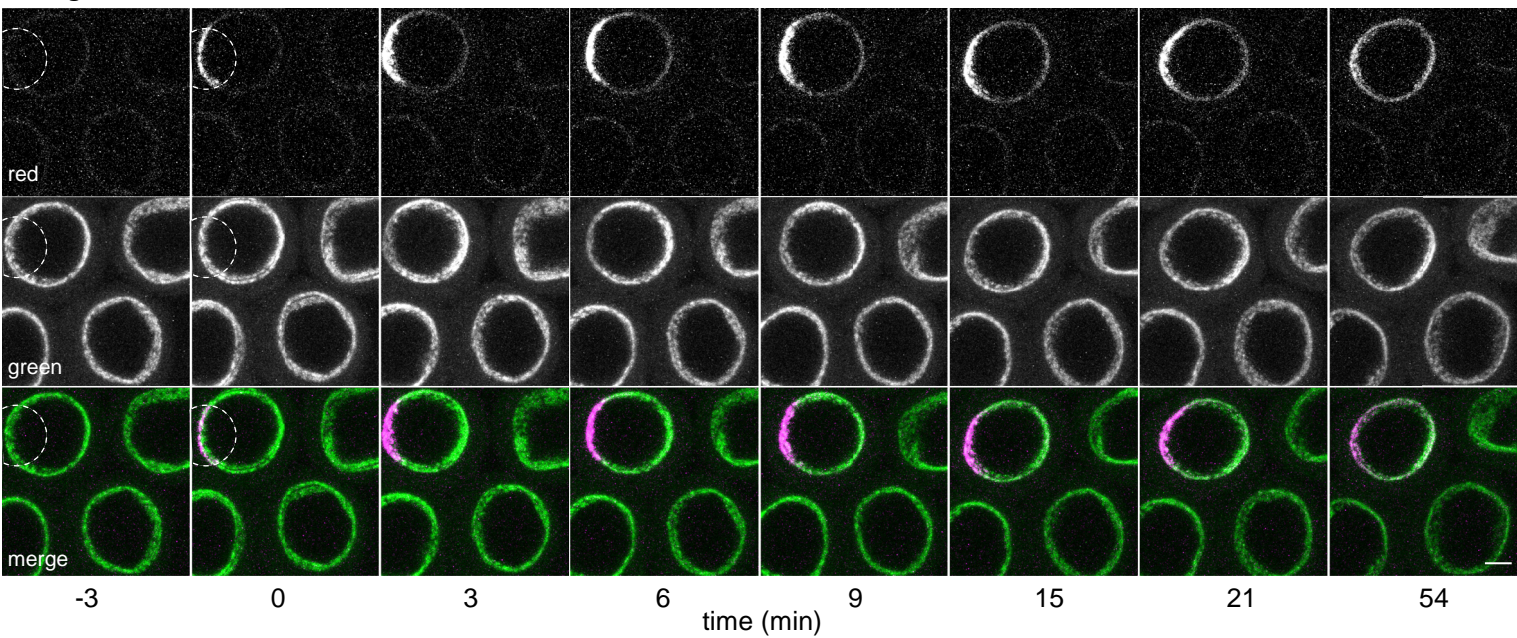

## **B**

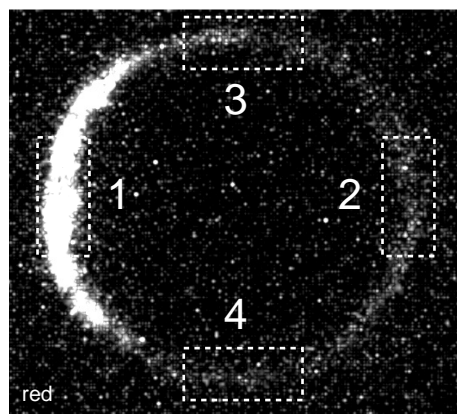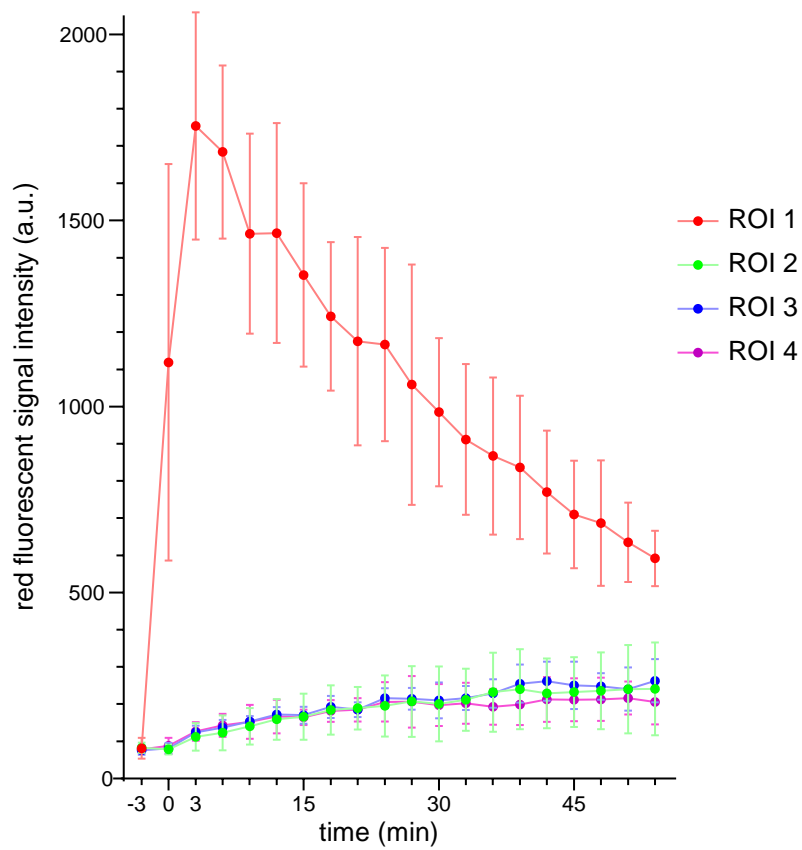

Supplement: S4 Fig — (A) Cysts with S6 spermatocytes expressing g-Mst27D-Dendra2 were analyzed by time-lapse imaging. The green initial fluorescence of Mst27D-Dendra2 in a region containing part of the NE (dashed circle) was photoconverted in one of the spermatocytes. Photoconversion was performed concomitant with acquisition of the z-stack at time 0. Redistribution of photoconverted red fluorescent Mst27D-Dendra2 was analyzed over time. Still frames display the green and red fluorescent Mst27D-Dendra2 signals, as well as their merge, as maximum intensity projections of 10 optical sections with 500 nm spacing. Scale bar = 5 μm. (B) For analysis of the dynamics of dispersal of photoconverted red fluorescent Mst27D-Dendra2, signal intensities in the red channel were quantified within four equal sized regions of interest (ROIs) (1–4). Average signal intensities (+/- s.d.) over time in the four ROIs are displayed (n = 5 S6 spermatocytes from distinct cysts). (PDF) [file pgen.1010837.s004.pdf]

**A**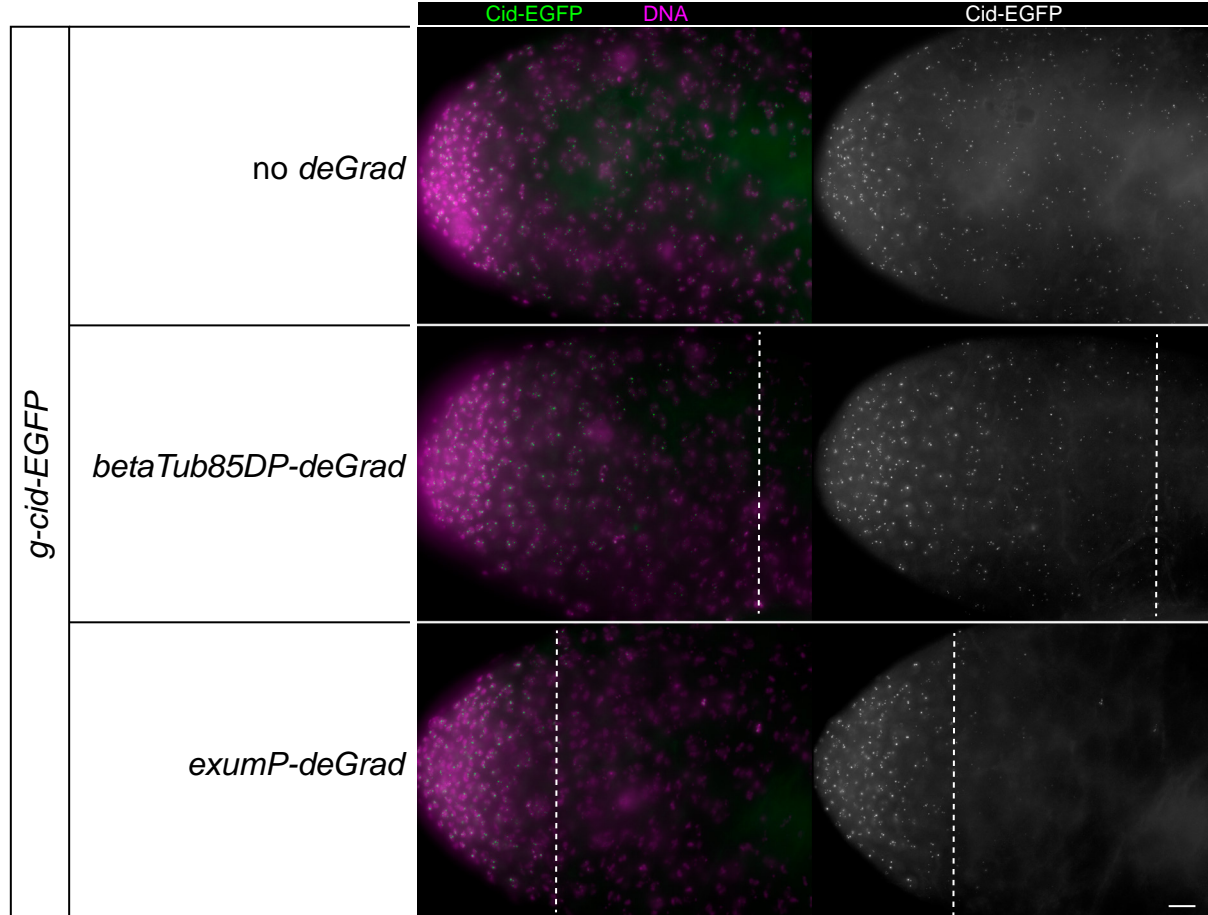**B**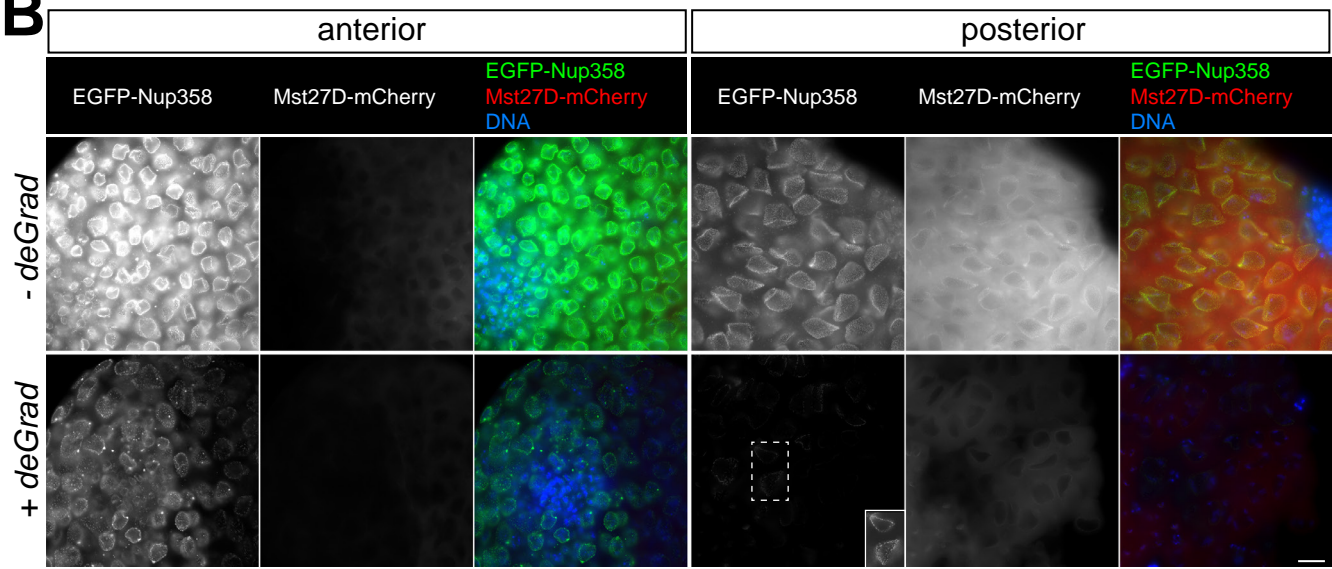

Supplement: S5 Fig — (A) Spermatocyte-specific degradation induced by the transgenes betaTub85DP-deGrad and exumP-deGrad. The functionality of these deGrad transgenes was evaluated in g-cid-EGFP testes, which express the centromere protein Cid/Cenp-A-EGFP, an EGFP fusion protein previously shown to be degraded efficiently by deGradFP [95]. Apical testis regions from whole mount preparations are displayed. While centromeric Cid-EGFP dot signals are present in all cells in the control (no deGrad), these signals are lost from spermatocytes distal from the dashed lines in g-cid-EGFP testes with betaTub85DP-deGrad or exumP-deGrad. (B) Spermatocyte-specific degradation of EGFP-Nup358 by exumP-deGrad in early pupal testes. Whole mount preparations were fixed and labeled with a DNA stain. The anterior regions with the initial stages of spermatogenesis and the posterior regions with late spermatocytes of representative -deGrad and +deGrad early pupal testes are shown with identical settings for imaging and display. A subregion (dashed rectangle) is shown with enhanced signals in the inset to reveal residual EGFP-Nup358 in late spermatocytes of +deGrad testes. Scale bars = 20 μm (PDF) [file pgen.1010837.s005.pdf]

**A**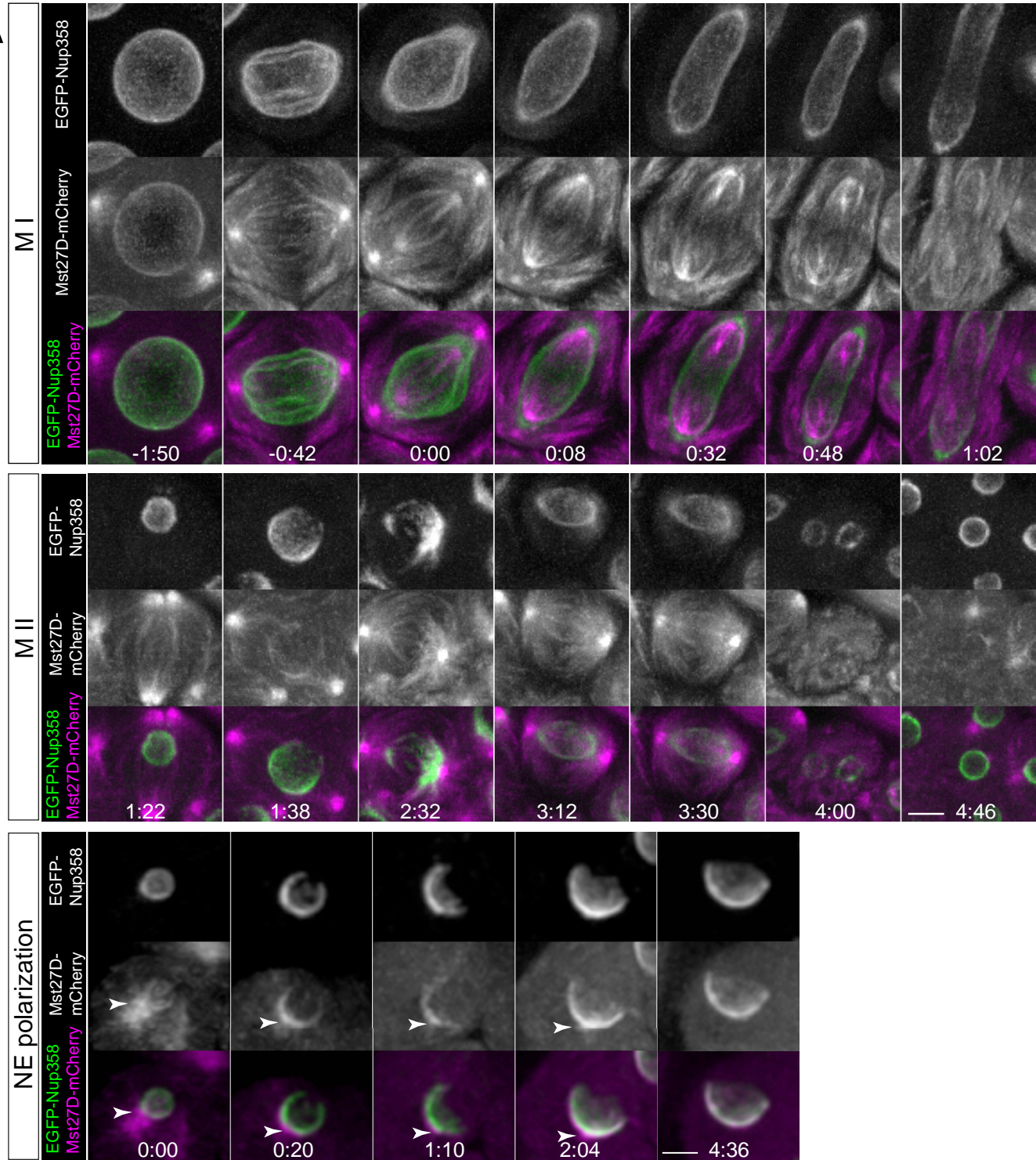**B**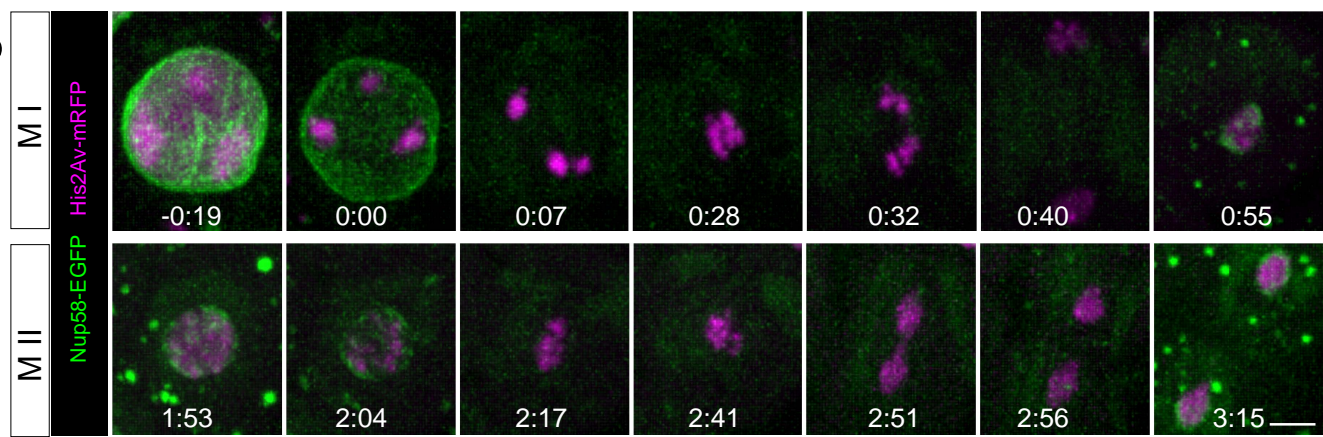

Supplement: S6 Fig — (A) Time-lapse imaging of cells expressing Mst27D-mCherry and EGFP-Nup358. Still frames display a spermatocyte from a first movie, documenting progression through meiosis I (M I) and meiosis II (M II), and an early post-meiotic spermatid from a second movie, documenting NE polarization. Time points (h:min) are indicated. In the first movie: NEBD I (0:00), metaphase I (0:32), anaphase I (0:48), telophase I (1:02), interkinesis (1:22, 1:38), NEBD II (2:32), metaphase II (3:12), anaphase II (3:30), telophase II (4:00) and post-meiotic interphase (4:46). In the second movie, t = 0:00 corresponds to early post-meiotic interphase, and the rapidly weaking Mst27D-mCherry enrichment on the centrosome is indicated (arrowhead). (B) Time-lapse imaging of a spermatocyte expressing Nup58-EGFP and histone H2Av-mRFP (His2Av-mRFP) during progression through meiosis I (M I) and meiosis II (M II). Time points (h:min) are indicated with t = 0 at the onset of NEBD I. Scale bars = 3 μm. (PDF) [file pgen.1010837.s006.pdf]

**A**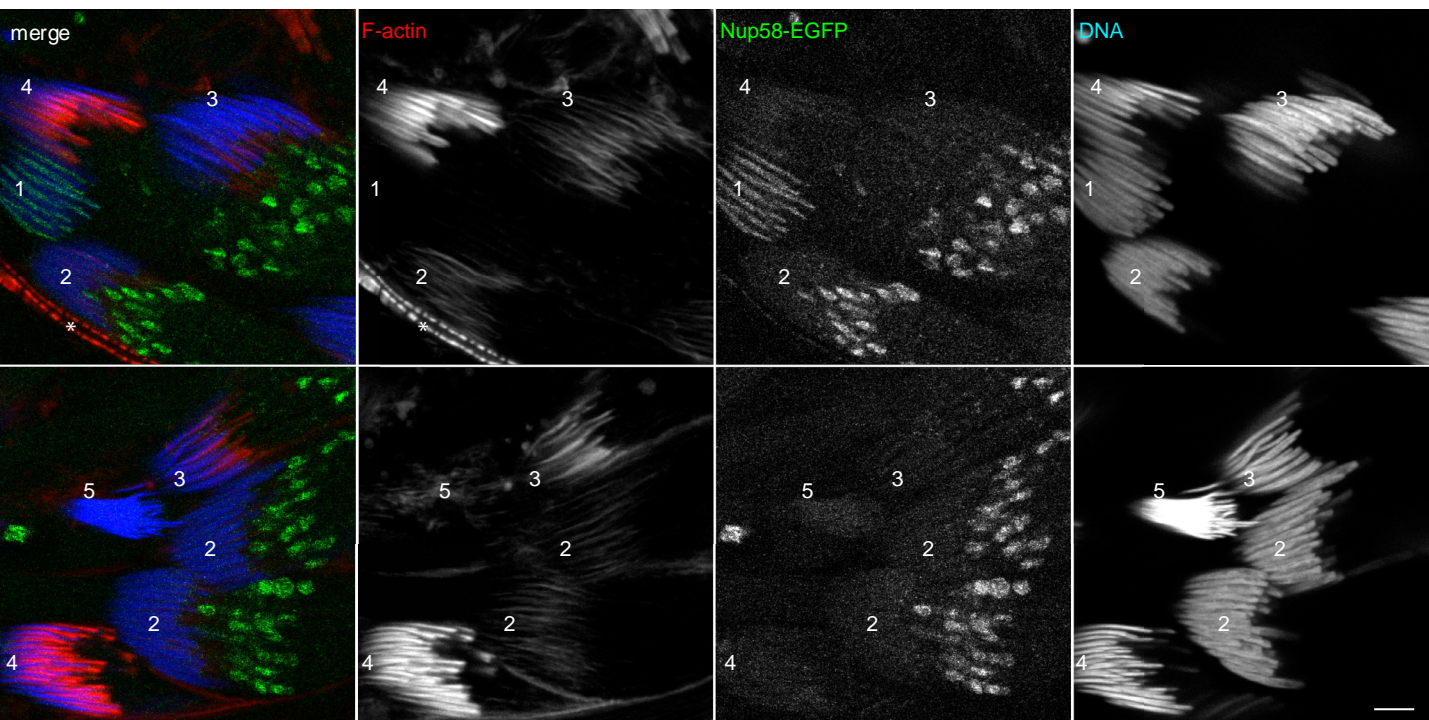**B**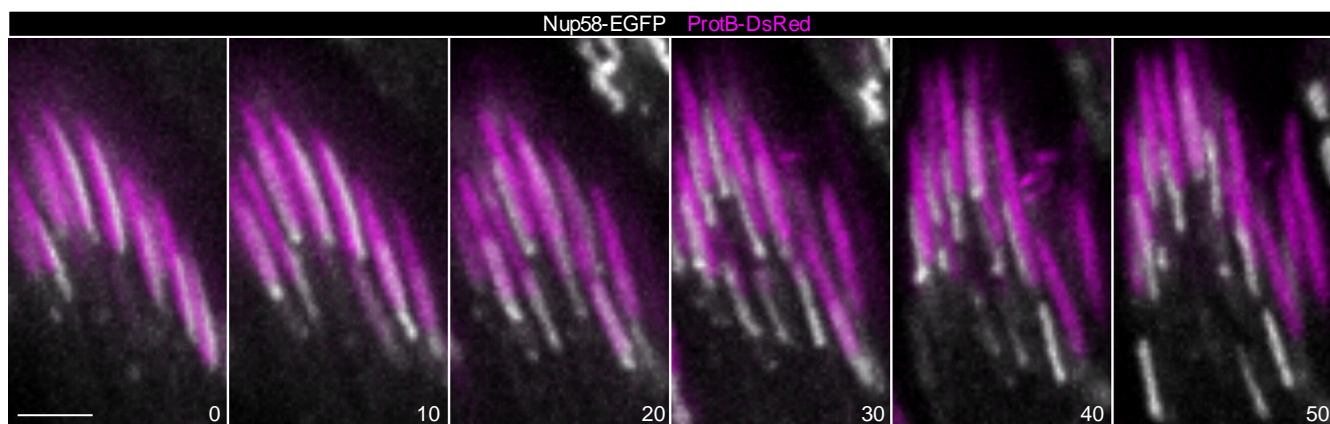**C**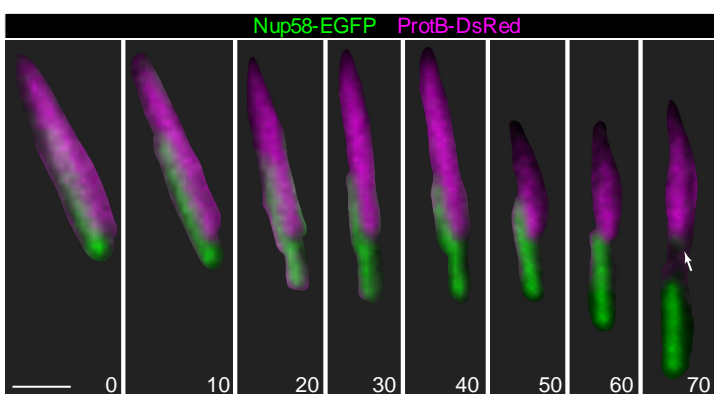**D**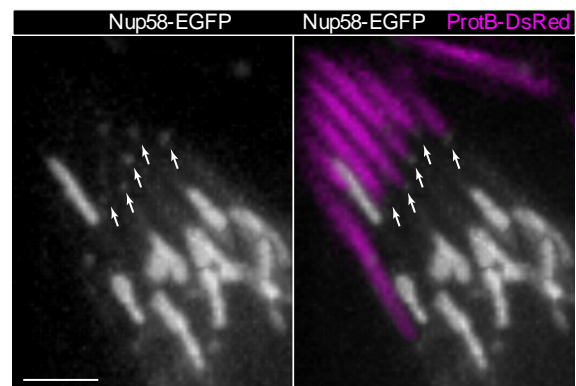**E**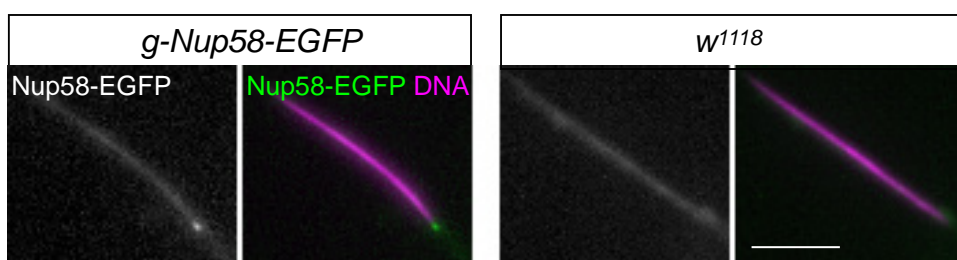

Supplement: S7 Fig — (A) Stage of NPC-NE shedding. Whole mount preparations of g-Nup58-EGFP testes were stained with fluorescent phalloidin to detect F-actin and with a DNA stain. Maximum intensity projections of testes regions with clustered spermatid nuclei of cysts during the stages of NPC-NE shedding and F-actin cone formation are displayed. Sperm head clusters of increasing ages are indicated with numbers: before shedding (1), early after shedding (2), and later stages (3–5). F-actin is not just prominent in the individualization cones but also in the muscle sheet around the testis tube (asterisk). (B-D) Dynamics of NPC-NE shedding. Spermatid cysts expressing g-Nup58-EGFP and g-ProtB-DsRed were analyzed by time-lapse imaging. (B) Still frames displaying single optical sections through the region with clustered elongated spermatid nuclei at the indicated time points (min). (C) Still frames displaying a single spermatid nucleus at the indicated time points (min). The original images acquired with spinning disk confocal microscopy were deconvolved before maximum intensity projection. (D) A weak Nup58-EGFP signal remains at the base of the elongated nuclei after completion of NPC-NE shedding (arrows). (E) The weak Nup58-EGFP signal at the base of the elongated nuclei is also detectable in mature sperm released from testis by squash preparation labeled with a DNA stain. The faint green dot signal is detected in g-Nup58-EGFP but not in w1118 control sperm. Scale bars = 5 μm (A,B,D,E) and 3 μm (C). (PDF) [file pgen.1010837.s007.pdf]

**A**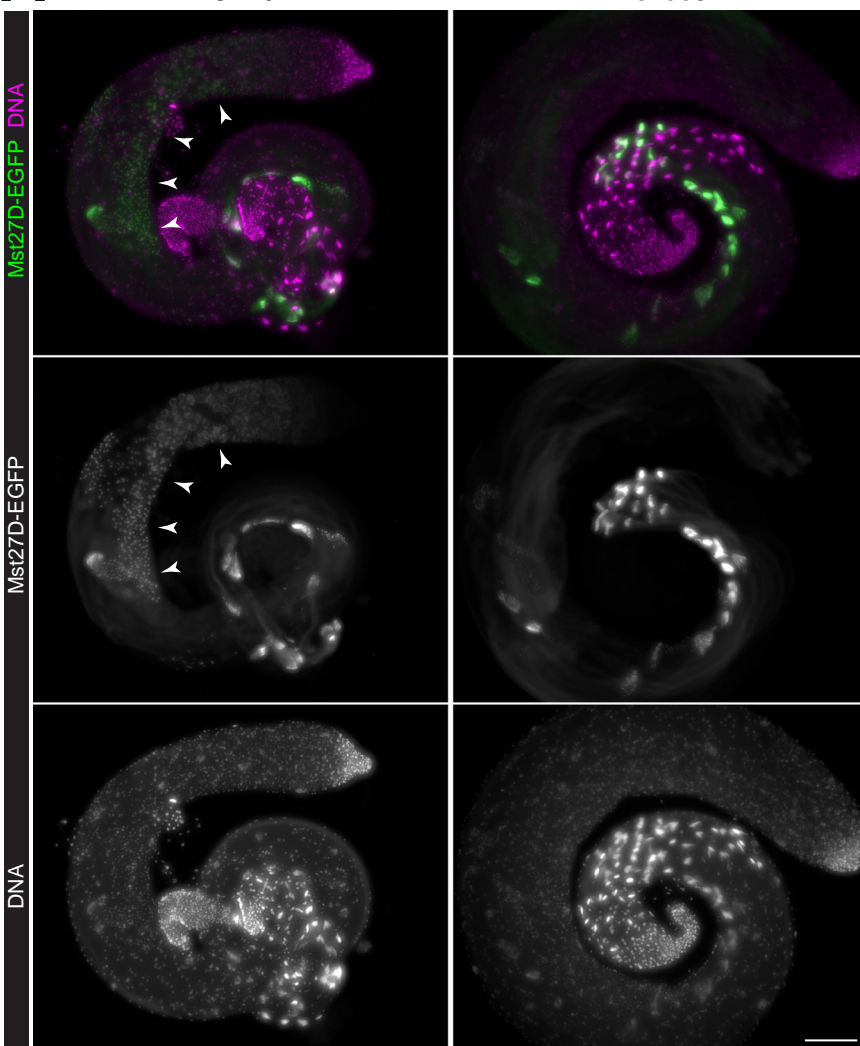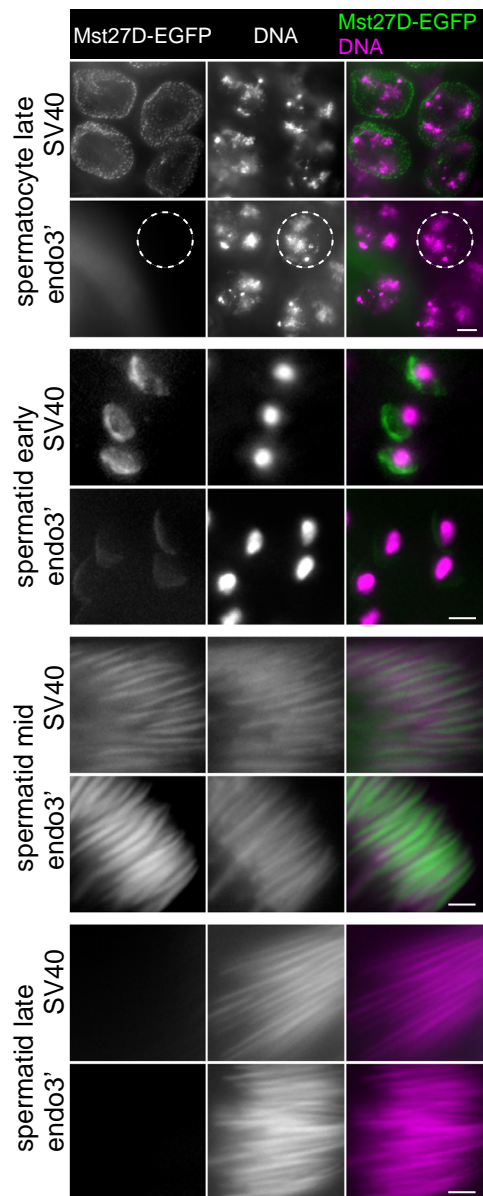**B**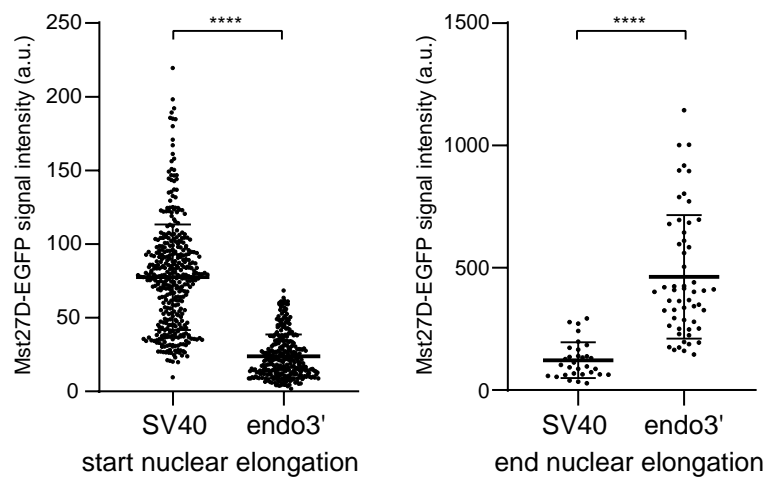

Supplement: S8 Fig — (A) Comparison of the pattern of Mst27D-EGFP accumulation driven by g-Mst27D-EGFP-endo3’ (endo3’), a transgene with downstream sequences identical to those at the endogenous Mst27D locus, and by g-Mst27D-EGFP (SV40), a transgene with SV40 terminator sequences replacing the endogenous Mst27D 3’ sequences. Whole mount preparations are displayed on the left (scale bar = 100 μm). Accumulation in spermatocytes (arrowheads) results with g-Mst27D-EGFP but not with g-Mst27D-EGFP-endo3’. High magnification views of single optical sections through nuclei at the indicated stages are displayed on the right (scale bars = 5 μm). Mst27D-EGFP is detectable in late spermatocyte nuclei in g-Mst27D-EGFP (SV40) testis but not in g-Mst27D-EGFP-endo3’ testis (dashed circle). (B) Quantification of Mst27D-EGFP signal intensity associated with the nuclei in spermatids at the start of nuclear elongation (left) and after completion of nuclear elongation (right) in the indicated genotypes. Individual nuclei and entire nuclear clusters were quantified at the early and late stage, respectively. Individual measurements and means (+/- s.d.) are displayed; n = 388 (SV40 early), 301 (endo3’ early), 31 (SV40 late) and 55 (endo3’ late). (PDF) [file pgen.1010837.s008.pdf]

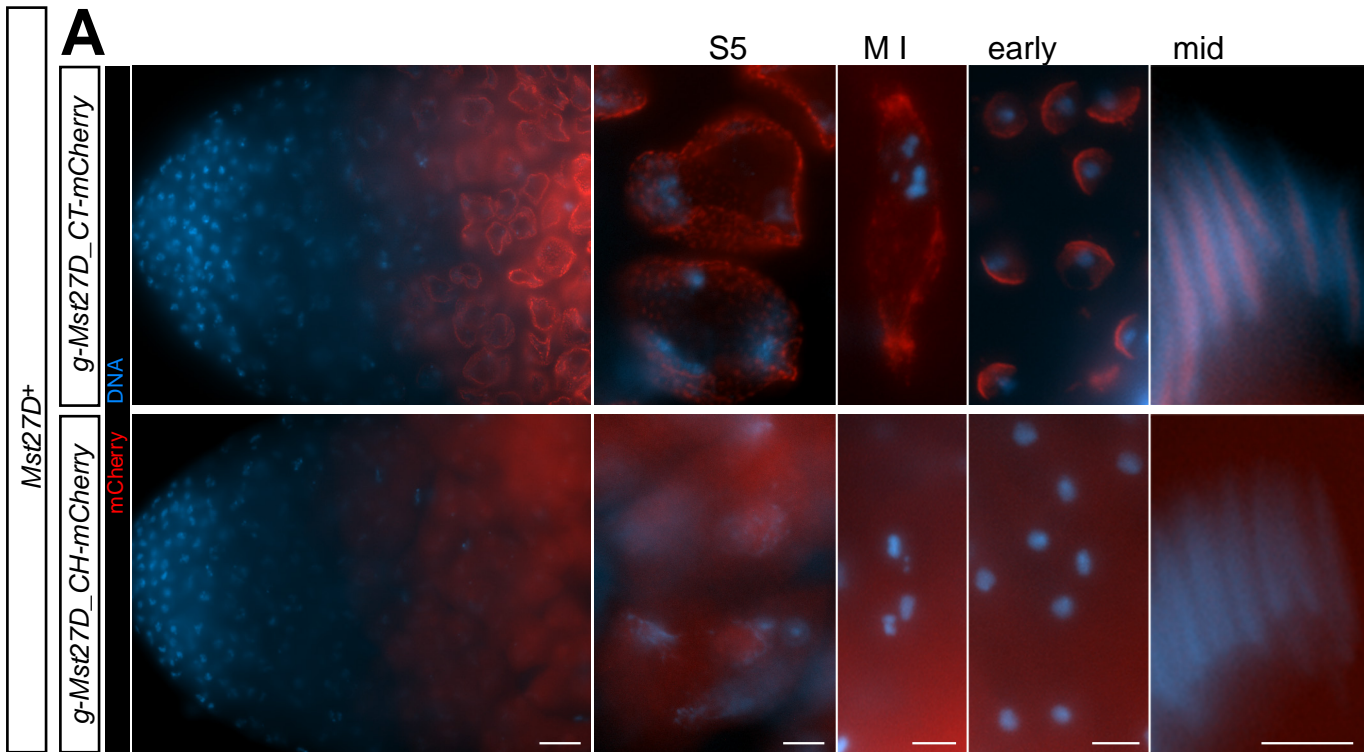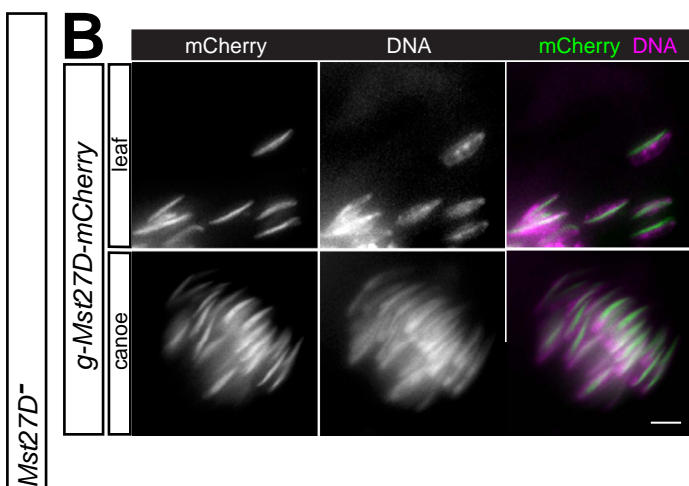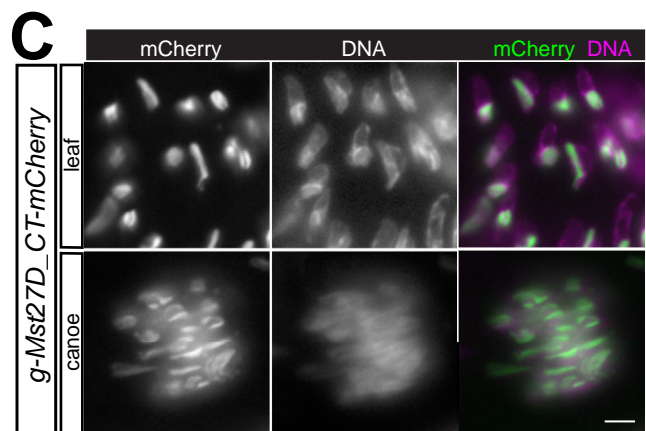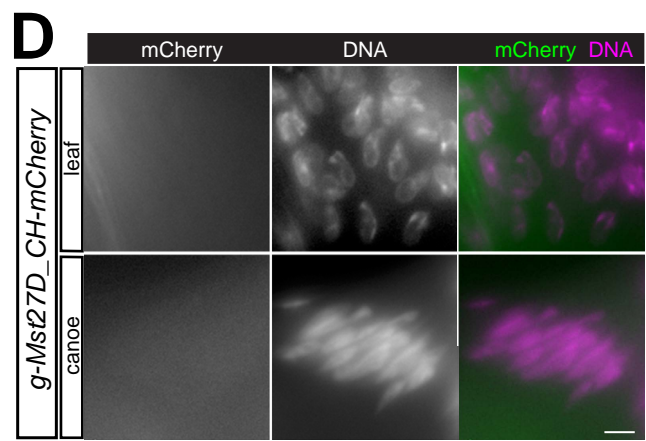

Supplement: S9 Fig — (A) Expression pattern and localization of Mst27D_CT-mCherry and Mst27D_CH-mCherry in testes with endogenous Mst27D function. Whole-mount preparations of testes isolated from transgenic males with g-Mst27D_CT-mCherry or g-Mst27D_CH-mCherry were fixed and labeled with a DNA stain. The apical regions of testes (left), as well as high magnification views of spermatocytes at the S5 stage (S5) and during prometaphase I (M I), as well as of spermatids early after NE polarization (early) and at the canoe stage (mid) are displayed. While Mst27D_CH-mCherry has a diffuse distribution, Mst27D_CT-mCherry localization corresponds to that of full length Mst27D-mCherry except during the meiotic stages, where the former displayed some enrichment also on the spindle envelope, while the latter is far more prominent on spindle MTs (S6 Fig). (B-D) Rescue of the nuclear elongation defect in Mst27D mutant spermatids by g-Mst27D-mCherry (B) but not by g-Mst27D_CT-mCherry (C) and g-Mst27D_CH-mCherry (D). The indicated transgenes were crossed into the Mst27DLL/ Df(2L)ade3 mutant background. Whole mount testes preparations were labeled with a DNA stain. Single optical sections display late spermatid nuclei at high magnification during the indicated stages. Scale bars = 20 μm for left column in (A) and 5 μm for all other images. (PDF) [file pgen.1010837.s009.pdf]

**A**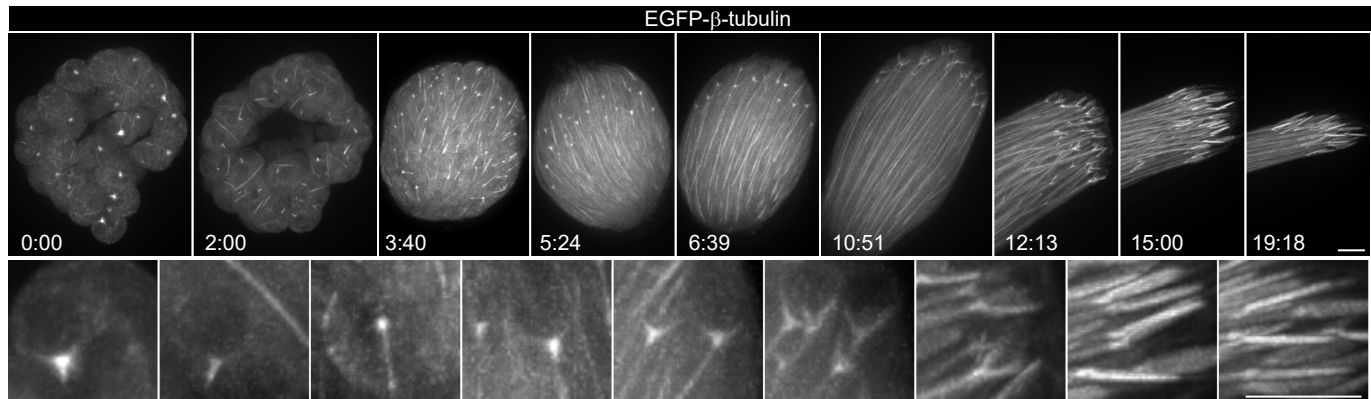**B**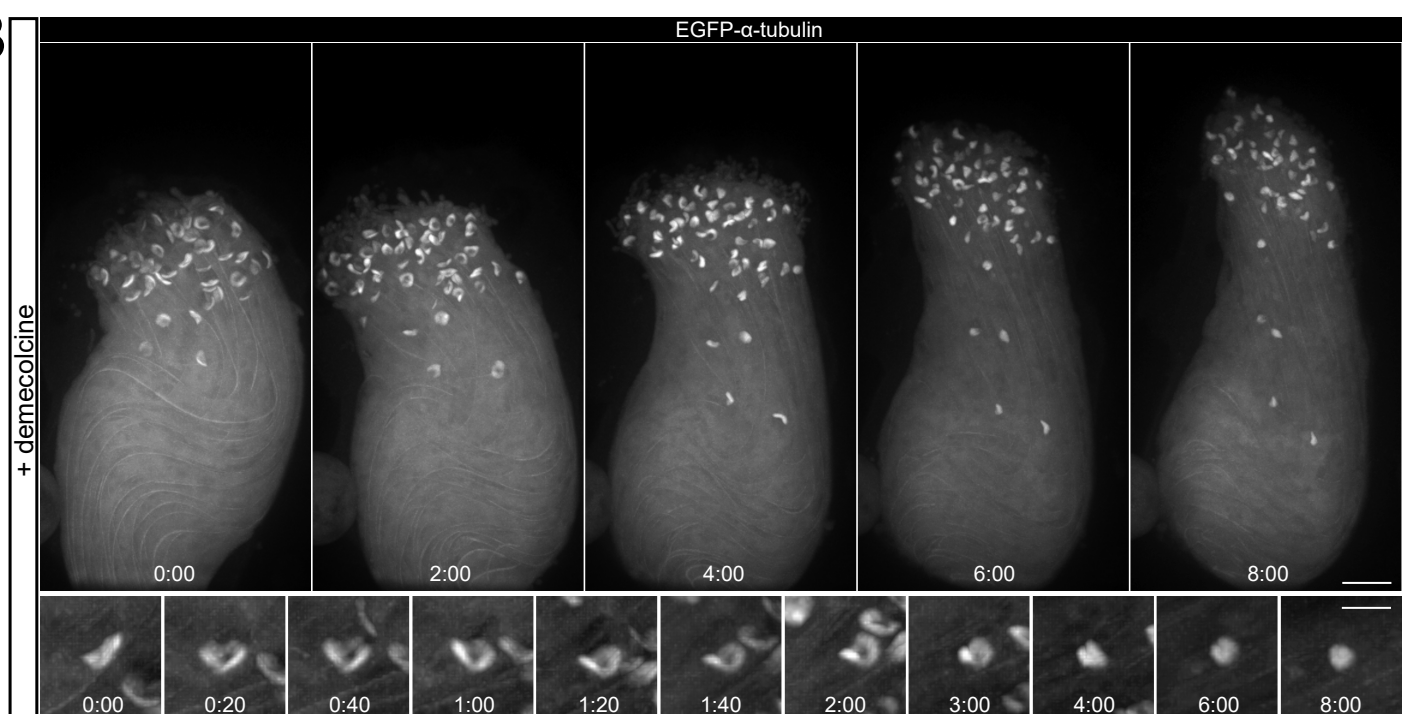**C**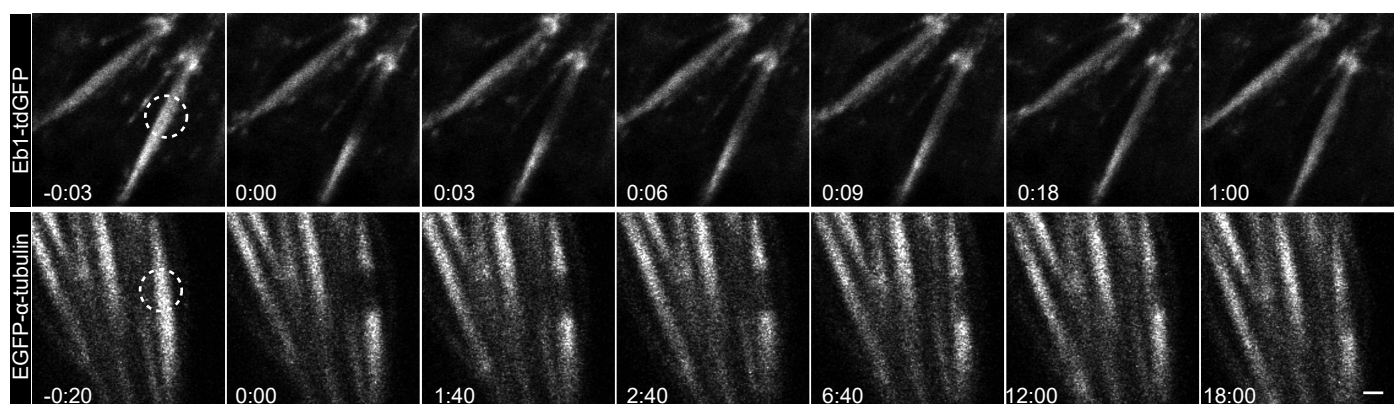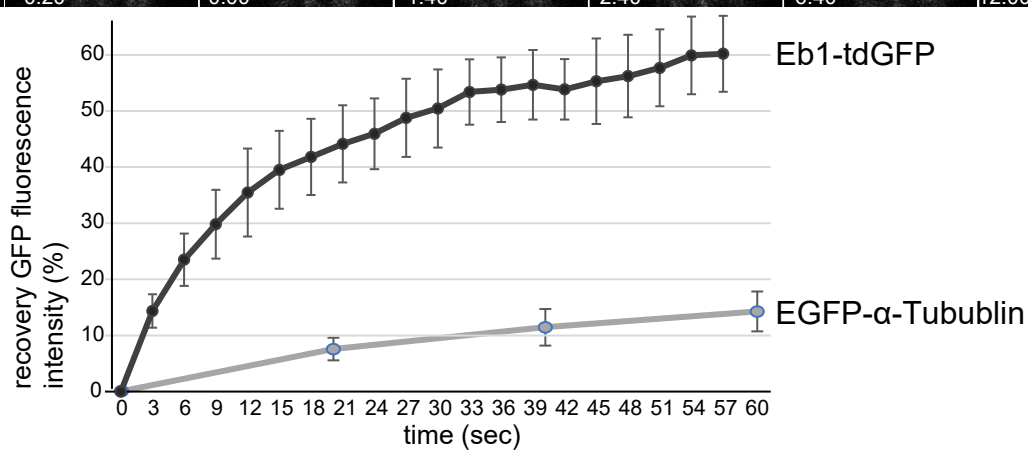

Supplement: S10 Fig — (A) Spermatid cysts expressing EGFP-β-tubulin were analyzed by time-lapse imaging to determine the temporal dynamics of cyst polarization, DC formation and nuclear elongation. Still frames (maximum intensity project of 30 optical sections with 500 nm spacing) from three distinct concatenated movies are displayed with time indicated (h:min; 0:00–2:00 from S1 Movie, 3:40–10:51 from S2 Movie, and 12:13–19:18 from S3 Movie). Time point t = 0 corresponds to end of M II. High magnification views of regions with basal body and DC-MTs for each time point are shown in bottom panel. (B) Effects of demecolcine on DC-MTs and nuclear elongation. Demecolcine was added before the start of time-lapse imaging of a spermatid cyst expressing EGFP-α-tubulin. A complete cyst (top) and high magnification views of a tracked spermatid nucleus (bottom) are displayed with time (h:min) indicated. (C) MT dynamics in the DC. Spermatids expressing either Eb1-tdGFP (top) or EGFP-α-tubulin (bottom) were used for fluorescence recovery after photobleaching (FRAP) experiments. Signals within in a central region of the DC-MTs were bleached and signal recovery was monitored at intervals of 3 seconds in case of Eb1-tdGFP and 20 seconds in case of EGFP-α-tubulin. Still frames of maximum intensity projections (three optical z-sections with 0.5 μm spacing) illustrate recovery at the indicated time points (min:sec). The diagram (bottom) represents extent of signal recovery over time. Average signal intensity before and immediately after bleaching were set to 100% and 0%, respectively. Mean values (+/- s.d.) are displayed; n = 6 cysts (Eb1-tdGFP) and 3 cysts (EGFP-α-tubulin). Scale bars = 10 μm (A and B, top), 4 μm (B, bottom) and 3 μm (C). (PDF) [file pgen.1010837.s010.pdf]

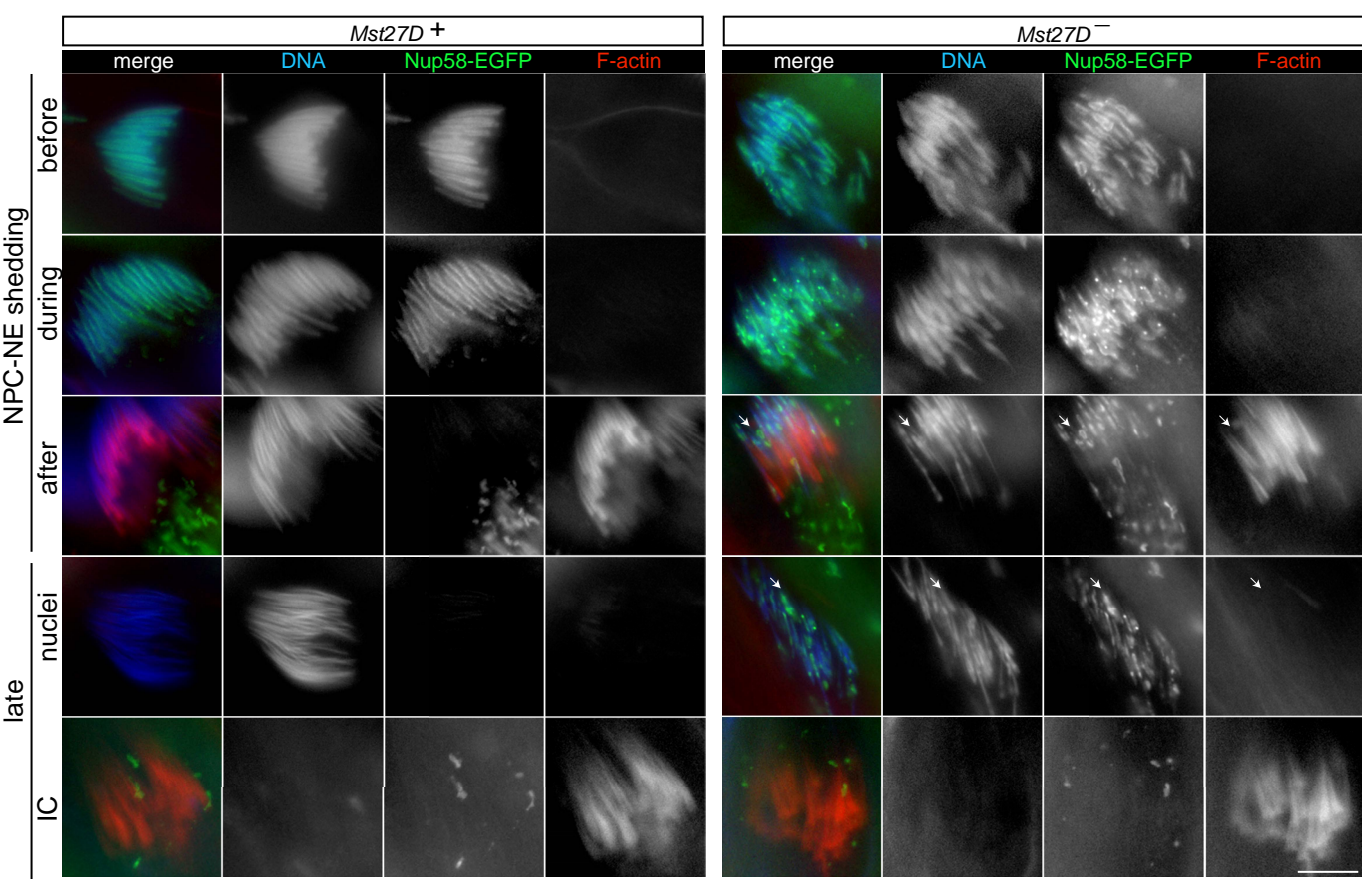

Supplement: S11 Fig — Testes expressing Nup58-EGFP in a background with Mst27D function (Mst27+) or without (Mst27D-) were used for whole mount preparations that were labeled with a DNA stain and with fluorescent phalloidin to reveal F-actin. Residual Nup58-EGFP in the NE of nuclei in late Mst27D mutant spermatids is indicated (arrows). Scale bar = 10 μm. (PDF) [file pgen.1010837.s011.pdf]

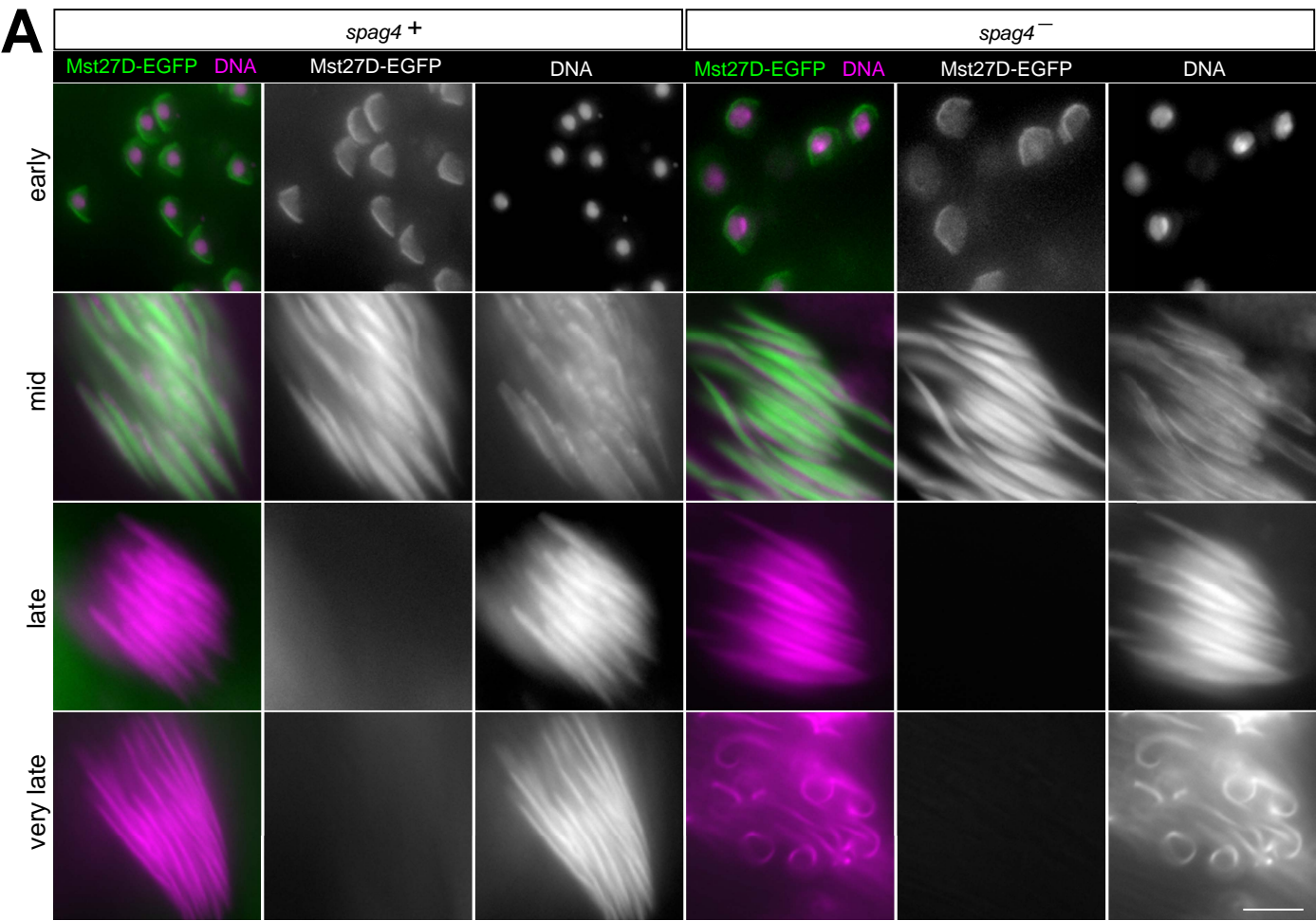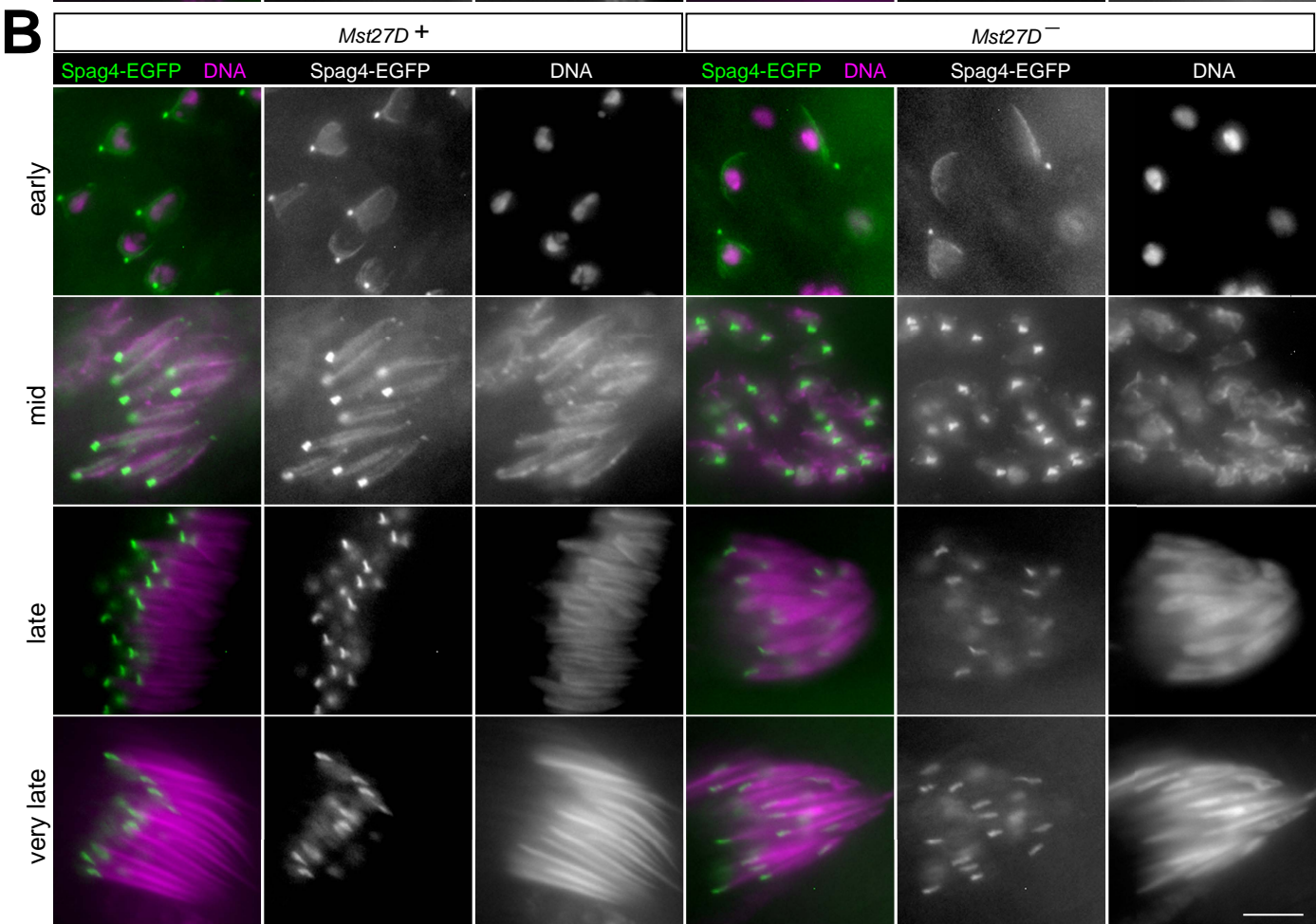

Supplement: S12 Fig — (A) Spag 4 is not required for normal Mst27D-EGFP localization in spermatids. Testes expressing g-Mst27D-EGFP in a background with spag4+ function (spag4+) or in spag41/spag46 mutants (spag4-) were used for whole mount preparations that were labeled with a DNA stain. Single optical sections display regions from the clustered spermatid nuclei at high magnification. (B) Mst27D is not required for normal Spag4-EGFP localization in spermatids. Testes expressing g-spag4-EGFP in a background with Mst27D+ function (Mst27D+) or in Mst27Dcc/ Df(2L)ade3 mutants (Mst27D-) were used for whole mount preparations that were labeled with a DNA stain. Single optical sections display regions from the clustered spermatid nuclei at high magnification. Scale bars = 5 μm. (PDF) [file pgen.1010837.s012.pdf]
