# Supplementary material for: Nuclear elongation during spermiogenesis depends on physical linkage of nuclear pore complexes to bundled microtubules by Drosophila Mst27D
Source: PLoS Genet. 2023 Jul 10;19(7):e1010837. doi: 10.1371/journal.pgen.1010837 (PMC10359004; doi:10.1371/journal.pgen.1010837)
Supplement: S2 Table — (PDF) [file pgen.1010837.s021.pdf]

S2 Table. Source of mutant alleles and transgenes.

genotype	source
w*; P{w^{+}, g-His2Av-mRFP} II.2	[1]
w*; P{w^{+}, g-His2Av-mRFP} III.1	[1]
w*; P{w^{+}, exumP-mCherry}att22A/ CyO	this work
y w*; wg^{Sp}/ CyO; P{w^{+}, Tpl94D-mRFP-1xFlag}SK3/ TM6B, Tb	[2], Kyoto Drosophila Stock Center #109817
w^{1118}; P{w^{8}, ProtB-DsRed-M1}50A (III)	[3], kindly provided by John Belote (Syracuse University, New York, USA)
w*; P{w^{+}, Ubq11-EGFP-alphaTub84B}/ CyO	[4], kindly provided by Renata Basto (Institute Curie, Paris, France)
w*; P{w^{+}, UbiP-GFP^{S65T}-betaTub56D} 17-1 (II)	[5], Kyoto Drosophila Stock Center #109603
y w*; Eb1-tdGFP (knock-in)	[6], kindly provided by José C. Pastor-Pareja (Tsinghua University, Beijing, PR China) and Renata Basto
w*; P{w^{+}, g-Nup58-EGFP}35B (12.4)/ CyO	[7]
w*; P{w^{+}, g-Nup58-EGFP} III.12	[7]
w*; P{w^{+}, g-Mst27D-mCherry} II.3/ CyO	this work
w*; P{w^{+}, g-Mst27D-mCherry} III.7/ TM3, Ser	this work
w*; P{w^{+}, g-Mst27D_CH-mCherry} III.1/ TM3, Ser	this work
w*; P{w^{+}, g-Mst27D_CT-mCherry} III.1/ TM3, Ser	this work
w*; P{w^{+}, g-Mst27D-Dendra2} III.1/ TM3, Ser	this work
w^{1118}; P{w^{+}, g-Mst27D-EGFP}	[8], kindly provided by Detlev Buttgereit (University of Marburg, Marburg, Germany)
w*; P{w^{+}, g-Mst27D-EGFP_endo3'} III.1/ TM3, Ser	this work
yw*; Mst27D^{cc1-4}/ CyO	this work
y* w*; PBac{SAstopDsRed}LL01793 P{ry^{+}, neoFRT}40A P{w^{+}, FRT(w^{hs})}G13 cn bw/ CyO, S* bw	[9], Kyoto Drosophila Stock Center #140463
w*; Df(2L)ade3/ CyO, P{w^{+}, Dfd-YFP} (deletes Mst27D)	Bloomington Drosophila stock center (BDSC) #6709
w^{1118}; emGFP-Nup358, PBac{y[+mDint2]=vas-Cas9}VK00027	[10], kindly provided by Bernhard Hampoelz (EMBL, Heidelberg, Germany)
w*; P{w^{+}, g-EGFP-Nup358} attP40	this work
w^{1118}; P{w^{+}, FMRFa-EGFP.Tv}3, P{w^{+}, UAS-myr-mRFP}2, Nup358^{12A002}/ TM6B, P{w^{+}, Dfd-EYFP}3, Sb Tb ca	[11], BDSC #59396
w^{1118}; PBac{w^{+}, IT.GAL4}Nup358$^{0175-G4}$/ TM6B, Tb	[12], BDSC #62684
w*; P{w^{+}, spag4-GFP} 3	[13], BDSC #29975
w*; TI{w^{+}, TI}spag4^{1}/ CyO	[13], BDSC #29977
w*; TI{w^{+}, TI}spag4^{6}/ CyO	[13], BDSC #29978
w*; P{w^{+}, exumP-NSlmb-vhhGFP4}attP40	[14]
w*; P{w^{+}, betaTub85DP-NSlmb-vhhGFP4}attP40	[15]
w*; P{w^{+}, g-cid-EGFP} III.2	[1]
w*; cid^{T12-1}/ CyO, P{ry+, ftz-lacZ}	[16]

w; cid^{T22-4}/ CyO, P{ry+, ftz-lacZ}*	[16]

References

1. Schuh M, Lehner CF, Heidmann S. Incorporation of Drosophila CID/CENP-A and CENP-C into centromeres during early embryonic anaphase. Current biology : CB. 2007; 17:237–43. Epub 2007/01/16. doi: 10.1016/j.cub.2006.11.051.

2. Kimura S. The Nap family proteins, CG5017/Hanabi and Nap1, are essential for Drosophila spermiogenesis. FEBS letters. 2013; 587:922–9. Epub 2013/02/20. doi: 10.1016/j.febslet.2013.02.019 PMID: 23454210.

3. Manier MK, Belote JM, Berben KS, Novikov D, Stuart WT, Pitnick S. Resolving mechanisms of competitive fertilization success in Drosophila melanogaster. Science. 2010; 328:354–7. doi: 10.1126/science.1187096 PMID: 20299550.

4. Dobbelaere J, Josue F, Suijkerbuijk S, Baum B, Tapon N, Raff J. A genome-wide RNAi screen to dissect centriole duplication and centrosome maturation in Drosophila. PLoS biology. 2008; 6:e224. doi: 10.1371/journal.pbio.0060224 PMID: 18798690.

5. Inoue YH, Savoian MS, Suzuki T, Mathe E, Yamamoto MT, Glover DM. Mutations in orbit/mast reveal that the central spindle is comprised of two microtubule populations, those that initiate cleavage and those that propagate furrow ingression. The Journal of cell biology. 2004; 166:49–60. doi: 10.1083/jcb.200402052 PMID: 15240569.

6. Sun T, Song Y, Dai J, Mao D, Ma M, Ni J-Q, et al. Spectraplakin Shot Maintains Perinuclear Microtubule Organization in Drosophila Polyploid Cells. Developmental cell. 2019; 49:731-747.e7. Epub 2019/04/18. doi: 10.1016/j.devcel.2019.03.027 PMID: 31006649.

7. Radermacher PT, Myachina F, Bosshardt F, Pandey R, Mariappa D, Muller HA, et al. O-GlcNAc reports ambient temperature and confers heat resistance on ectotherm development. Proceedings of the National Academy of Sciences of the United States of America. 2014; 111:5592–7. doi: 10.1073/pnas.1322396111 PMID: 24706800.

8. Gärtner SMK, Hundertmark T, Nolte H, Theofel I, Eren-Ghiani Z, Tetzner C, et al. Stage-specific testes proteomics of Drosophila melanogaster identifies essential proteins for male fertility. Eur J Cell Biol. 2019; 98:103–15. Epub 2019/01/17. doi: 10.1016/j.ejcb.2019.01.001 PMID: 30679029.

9. Schuldiner O, Berdnik D, Levy JM, Wu JS, Luginbuhl D, Gontang AC, et al. piggyBac-based mosaic screen identifies a postmitotic function for cohesin in regulating developmental axon pruning. Developmental cell. 2008; 14:227–38. doi: 10.1016/j.devcel.2007.11.001 PMID: 18267091.

10. Hampoelz B, Schwarz A, Ronchi P, Bragulat-Teixidor H, Tischer C, Gaspar I, et al. Nuclear Pores Assemble from Nucleoporin Condensates During Oogenesis. Cell. 2019; 179:671-686.e17. doi: 10.1016/j.cell.2019.09.022 PMID: 31626769.

11. Bivik C, Bahrampour S, Ulvklo C, Nilsson P, Angel A, Fransson F, et al. Novel Genes Involved in Controlling Specification of Drosophila FMRFamide Neuropeptide Cells. Genetics. 2015; 200:1229–44. Epub 2015/06/18. doi: 10.1534/genetics.115.178483 PMID: 26092715.

12. Gohl DM, Silies MA, Gao XJ, Bhalerao S, Luongo FJ, Lin C-C, et al. A versatile in vivo system for directed dissection of gene expression patterns. Nat Methods. 2011; 8:231–7. doi: 10.1038/nmeth.1561 PMID: 21473015.

13. Kracklauer MP, Wiora HM, Deery WJ, Chen X, Bolival, B., Jr., Romanowicz D, et al. The Drosophila SUN protein Spag4 cooperates with the coiled-coil protein Yuri Gagarin to maintain association of the basal body and spermatid nucleus. Journal of cell science. 2010; 123:2763–72. doi: 10.1242/jcs.066589 PMID: 20647369.

14. Chaurasia S. Dynamics of the Meiotic Division in Drosophila Males. PhD thesis, University of Zurich. 2017.

15. Sun MS, Weber J, Blattner AC, Chaurasia S, Lehner CF. MNM and SNM maintain but do not establish achiasmate homolog conjunction during Drosophila male meiosis. PLoS genetics. 2019; 15:e1008162. doi: 10.1371/journal.pgen.1008162 PMID: 31136586.
16. Blower MD, Daigle T, Kaufman T, Karpen GH. Drosophila CENP-A mutations cause a BubR1-dependent early mitotic delay without normal localization of kinetochore components. PLoS genetics. 2006; 2:e110. Epub 2006/07/15. doi: 10.1371/journal.pgen.0020110.
