# Supplementary material for: Nuclear elongation during spermiogenesis depends on physical linkage of nuclear pore complexes to bundled microtubules by Drosophila Mst27D
Source: PLoS Genet. 2023 Jul 10;19(7):e1010837. doi: 10.1371/journal.pgen.1010837 (PMC10359004; doi:10.1371/journal.pgen.1010837)
Supplement: S3 Table — (PDF) [file pgen.1010837.s022.pdf]

S3 Table. Genotypes used in experiments.

figure	label on figure	genotype
4A	EGFP-Nup358, Mst27D-mCherry	P{w^{+}, g-EGFP-Nup358} attP40; PBac{w^{+}, IT.GAL4}Nup358$^{0175\text{-}G4}$, P{w^{+}, g-Mst27D-mCherry} III.7
4C	- deGrad	w*; P{w^{+}, g-EGFP-Nup358} attP40/ +; PBac{w^{+}, IT.GAL4}Nup358$^{0175\text{-}G4}$, P{w^{+}, g-Mst27D-mCherry} III.7/ P{w^{+}, FMRFa-EGFP.Tv}3, P{w^{+}, UAS-myr-mRFP}2, Nup358^{12A002}
	+ deGrad	w*; P{w^{+}, g-EGFP-Nup358} attP40/ P{w^{+}, exumP-NSlmb-vhhGFP4}attP40; PBac{w^{+}, IT.GAL4}Nup358$^{0175\text{-}G4}$, P{w^{+}, g-Mst27D-mCherry} III.7/ P{w^{+}, FMRFa-EGFP.Tv}3, P{w^{+}, UAS-myr-mRFP}2, Nup358^{12A002}
5A	emGFP-Nup358, Mst27D-mCherry	w*; P{w^{+}, g-Mst27D-mCherry} II.3; emGFP-Nup358, PBac{y[$^{+}$mDint2]=vas-Cas9}VK00027
5D	Mst27D^{-}	w*; Mst27D$^{cc1\text{-}4}$/ Df(2L)ade3
	Mst27D^{+}	w^{1118}
6B	w1118	w^{1118}
	Df/+	w*; Df(2L)ade3/ CyO, S* bw
	Mst27D[LL]/+	w*; PBac{SAstopDsRed}LL01793 P{ry^{+}, neoFRT}40A P{w^{+}, FRT(whs)}G13 cn bw/ CyO, P{w^{+}, Dfd-YFP}
	Mst27D[LL]/Df	w*; PBac{SAstopDsRed}LL01793 P{ry^{+}, neoFRT}40A P{w^{+}, FRT(whs)}G13 cn bw/ Df(2L)ade3
	Mst27D[LL]/Df + g-Mst27D-EGFP	w*; PBac{SAstopDsRed}LL01793 P{ry^{+}, neoFRT}40A P{w^{+}, FRT(whs)}G13 cn bw/ Df(2L)ade3; P{w^{+}, g-Mst27D-EGFP}/ +
6C	Mst27D[LL]/Df + g-Mst27D-mCherry	w*; PBac{SAstopDsRed}LL01793 P{ry^{+}, neoFRT}40A P{w^{+}, FRT(whs)}G13 cn bw/ Df(2L)ade3; P{w^{+}, g-Mst27D-mCherry} III.7/ +
	Mst27D[LL]/Df + g-Mst27D_CH-mCherry	w*; PBac{SAstopDsRed}LL01793 P{ry^{+}, neoFRT}40A P{w^{+}, FRT(whs)}G13 cn bw/ Df(2L)ade3; P{w^{+}, g-Mst27D_CH-mCherry} III.1/ +
	Mst27D[LL]/Df + g-Mst27D_CT-mCherry	w*; PBac{SAstopDsRed}LL01793 P{ry^{+}, neoFRT}40A P{w^{+}, FRT(whs)}G13 cn bw/ Df(2L)ade3; P{w^{+}, g-Mst27D_CT-mCherry} III.1/ +
6D	Mst27D[cc]	yw*; Mst27D$^{cc1\text{-}4}$
6E	Mst27D^{-}	w*; P{w^{+}, g-EGFP-Nup358} attP40/ +; Mst27D$^{cc1\text{-}4}$/ PBac{SAstopDsRed}LL01793 P{ry^{+}, neoFRT}40A P{w^{+}, FRT(whs)}G13 cn bw
	Mst27D^{+}	w*; P{w^{+}, g-EGFP-Nup358} attP40/ +; Mst27D$^{cc1\text{-}4}$/ +
6F	Mst27D^{-}	w*; P{w^{+}, Ubq11-EGFP-alphaTub84B}, Mst27D$^{cc1\text{-}4}$/ Df(2L)ade3; P{w^{+}, Tpl94D-mRFP-1xFlag}SK3/ +
	Mst27D^{+}	w*; P{w^{+}, Ubq11-EGFP-alphaTub84B}, Mst27D$^{cc1\text{-}4}$/ +; P{w^{+}, Tpl94D-mRFP-1xFlag}SK3/ +
6G	Mst27D^{-}	w*; P{w^{+}, Ubq11-EGFP-alphaTub84B}, Mst27D$^{cc1\text{-}4}$/ Df(2L)ade3; P{w8, ProtB-DsRed-M1}50A (III)/ +
	Mst27D^{+}	w*; P{w^{+}, Ubq11-EGFP-alphaTub84B}, Mst27D$^{cc1\text{-}4}$/ +; P{w8, ProtB-DsRed-M1}50A (III)/ +
7A	Mst27D^{+}	w*; P{w^{+}, Ubq11-EGFP-alphaTub84B}, Mst27D$^{cc1\text{-}4}$/ +; P{w^{+}, g-His2Av-mRFP} III.1/ +
7B	EGFP-α-tubulin, His2Av-mRFP	w*; P{w^{+}, Ubq11-EGFP-alphaTub84B}, P{w^{+}, g-His2Av-mRFP} II.2; MKRS/TM6B, Tb, Hu

7C	EGFP-β-tubulin, His2Av-mRFP	w*; P{w^{+}, UbiP-GFPS65T-betaTub56D} 17-1 (II), P{w^{+}, g-His2Av-mRFP} II.2
7D	EGFP-α-tubulin, Mst27D-mCherry	w*; P{w^{+}, Ubq11-EGFP-alphaTub84B}; P{w^{+}, g-Mst27D-mCherry} III.7
7E	Mst27D^{-}	w*; P{w^{+}, Ubq11-EGFP-alphaTub84B}, Mst27D^{cc1-4}/ Df(2L)ade3; P{w^{+}, g-His2Av-mRFP} III.1/ +
S4	green	w*; ; P{w^{+}, g-Mst27D-Dendra2} III.1
S5A	no deGrad	w*; cid^{T12-1} or cid^{T22-4}/ +; P{w^{+}, g-cid-EGFP-cid} III.2/ +
	betaTub85DP-deGrad	w*; cid^{T12-1} or cid^{T22-4}/ P{w^{+}, betaTub85DP-NSlmb-vhhGFP4}attP40; P{w^{+}, g-cid-EGFP-cid} III.2/ +
	exumP-deGrad	w*; cid^{T12-1} or cid^{T22-4}/ P{w^{+}, exumP-NSlmb-vhhGFP4}attP40; P{w^{+}, g-cid-EGFP-cid} III.2/ +
S5B	- deGrad	w*; P{w^{+}, g-EGFP-Nup358} attP40/ +; PBac{w^{+}, IT.GAL4}Nup358$^{0175-G4}$, P{w^{+}, g-Mst27D-mCherry} III.7/ P{w^{+}, FMRFa-EGFP.Tv}3, P{w^{+}, UAS-myr-mRFP}2, Nup358^{12A002}
	+ deGrad	w*; P{w^{+}, g-EGFP-Nup358} attP40/ P{w^{+}, exumP-NSlmb-vhhGFP4}attP40; PBac{w^{+}, IT.GAL4}Nup358$^{0175-G4}$, P{w^{+}, g-Mst27D-mCherry} III.7/ P{w^{+}, FMRFa-EGFP.Tv}3, P{w^{+}, UAS-myr-mRFP}2, Nup358^{12A002}
S6A	EGFP-Nup358, Mst27D-mCherry	w*; P{w^{+}, g-EGFP-Nup358} attP40/ +; PBac{w^{+}, IT.GAL4}Nup358$^{0175-G4}$, P{w^{+}, g-Mst27D-mCherry} III.7/ P{w^{+}, FMRFa-EGFP.Tv}3, P{w^{+}, UAS-myr-mRFP}2, Nup358^{12A002}
S6B	Nup58-EGFP, His2Av-mRFP	w*; P{w^{+}, g-Nup58-EGFP} 35B (12.4), P{w^{+}, g-His2Av-mRFP} II.2
S7A	Nup58-EGFP	w*; P{w^{+}, g-Nup58-EGFP}35B (12.4)
S7B-D	Nup58-EGFP, ProtB-DsRed	w*; P{w^{+}, g-Nup58-EGFP}35B (12.4); P{w8, ProtB-DsRed-M1}50A (III)
S7E	Nup58-EGFP	w*; P{w^{+}, g-Nup58-EGFP}35B (12.4), P{w^{+}, g-His2Av-mRFP} II.2
	w1118	w^{1118}
S8	SV40	w^{1118}; ; P{w^{+}; g-Mst27D-EGFP}
	endo3'	w*; ; P{w^{+}, g-Mst27D-EGFP_endo3'} III.1
S9A	g-Mst27D_CT-mCherry	w*; ; P{w^{+}, g-Mst27D_CT-mCherry} III.1
	g-Mst27D_CH-mCherry	w*; ; P{w^{+}, g-Mst27D_CH-mCherry} III.1
S9B	g-Mst27D-mCherry	PBac{SAstopDsRed}LL01793 P{ry^{+}, neoFRT}40A P{w^{+}, FRT(whs)}G13 cn bw/ Df(2L)ade3; P{w^{+}, g-Mst27D-mCherry} III.7/ +
S9C	g-Mst27D_CT-mCherry	PBac{SAstopDsRed}LL01793 P{ry^{+}, neoFRT}40A P{w^{+}, FRT(whs)}G13 cn bw/ Df(2L)ade3; P{w^{+}, g-Mst27D_CT-mCherry} III.1/ +
S9D	g-Mst27D_CH-mCherry	PBac{SAstopDsRed}LL01793 P{ry^{+}, neoFRT}40A P{w^{+}, FRT(whs)}G13 cn bw/ Df(2L)ade3; P{w^{+}, g-Mst27D_CH-mCherry} III.1/ +
S10A	EGFP-β-tubulin	w*; P{w^{+}, UbiP-GFPS65T-betaTub56D} 17-1 (II)
S10B	EGFP-α-tubulin	w*; P{w^{+}, Ubq11-EGFP-alphaTub84B}; P{w^{+}, g-Mst27D-mCherry} III.7
S10C	Eb1-tdGFP	yw*; Eb1-tdGFP (knock-in)
	EGFP-α-tubulin	w*; P{w^{+}, Ubq11-EGFP-alphaTub84B}
S11	Mst27D^{+}	w*; PBac{SAstopDsRed}LL01793 P{ry^{+}, neoFRT}40A P{w^{+}, FRT(whs)}G13 cn bw/ +; P{w^{+}, g-Nup58-EGFP}III.12/ +
	Mst27D^{-}	w*; PBac{SAstopDsRed}LL01793 P{ry^{+}, neoFRT}40A P{w^{+}, FRT(whs)}G13 cn bw/ Df(2L)ade3; P{w^{+}, g-Nup58-EGFP}III.12/ +

S12A	Spag4^{+}	w*; TI{w^{+}, TI}spag4^{6}/ +; P{w^{+}, g-Mst27D-EGFP_endo3'} III.1/ +
	Spag4^{-}	w*; TI{w^{+}, TI}spag4^{6}/ TI{w^{+}, TI}spag4^{1}; P{w^{+}, g-Mst27D-EGFP_endo3'} III.1/ +
S12B	Mst27D^{+}	w*; Mst27D^{cc1-4}/ +; P{w^{+}, spag4-GFP} 3/ +
	Mst27D^{-}	w*; Mst27D^{cc1-4}/ Df(2L)ade3; P{w^{+}, spag4-GFP} 3/ +
S1 Movie		w*; P{w^{+}, g-EGFP-Nup358} attP40/ +; PBac{w^{+}, IT.GAL4}Nup358$^{0175-G4}$, P{w^{+}, g-Mst27D-mCherry} III.7/ P{w^{+}, FMRFa-EGFP.Tv}3, P{w^{+}, UAS-myr-mRFP}2, Nup358^{12A002}
S2 Movie		w*; P{w^{+}, g-Nup58-EGFP}35B (12.4); P{w8, ProtB-DsRed-M1}50A (III)
S3 Movie		w*; P{w^{+}, UbiP-GFPS65T-betaTub56D} 17-1 (II)
S4 Movie		w*; P{w^{+}, Ubq11-EGFP-alphaTub84B}, Mst27D^{cc1-4}/ +; P{w^{+}, g-His2Av-mRFP} III.1/ +
S5 Movie		w*; P{w^{+}, UbiP-GFPS65T-betaTub56D} 17-1 (II), P{w^{+}, g-His2Av-mRFP} II.2
S6 Movie		w*; P{w^{+}, Ubq11-EGFP-alphaTub84B}; P{w^{+}, g-Mst27D-mCherry} III.7
S7 Movie		w*; P{w^{+}, Ubq11-EGFP-alphaTub84B}, Mst27D^{cc1-4}/ Df(2L)ade3; P{w^{+}, g-His2Av-mRFP} III.1/ +
