# Supplementary material for: Nuclear elongation during spermiogenesis depends on physical linkage of nuclear pore complexes to bundled microtubules by Drosophila Mst27D
Source: PLoS Genet. 2023 Jul 10;19(7):e1010837. doi: 10.1371/journal.pgen.1010837 (PMC10359004; doi:10.1371/journal.pgen.1010837)
Supplement: S4 Table — (PDF) [file pgen.1010837.s023.pdf]

S4 Table. Synthetic DNA sequences.

name	sequence (5’-3’)
CL231	ATTAGCGGCCGCTTACTTGTACAGCTCGTCCATGC
CL298	TAATACGACTCACTATAGGGAGACAGTGGCGTCTGGCGGAAAA
CL299	TAATACGACTCACTATAGGGAGATCCGAGCCAGTTTACCCGCT
CL342	GAAGGACACTAGTCCATGGACCAACATGGTGAAGAGGGTAACAGCGAGCAAATACGATGTAGTCTCGGTCAAGACGCTGCTGATGTTTATCAACAGCAAACTGGATTGCGAATTGCGAGGCTTTGAGGATCTGAAAACAGGAGCAGTATATTGCCAACTGATGCACAGACTCTTTCCCAATTCGATACAAATTCATAAGGTCAAGTTTTATACGAACGACATCAGCGACTTTCAGTTAAATTTCCGGTTACTAAACACTTGCTTTCAAAAACTAAGAGTAACCGTTTATATGCCAGTGCATGAGTTAACTCTGGGACACAATCAAGTGGTCTTCTGCAATTGGATATACAAGTTCTATGAAGCAAACGACAAGGGGAACGAATACGATGCCAGAAAGGTGAGAAAAGGCTCGCCAATCGGTTTAGACAATAGTCGAGATCTGGACGG
CL343	GAGGACACTAGTTATAAGGTAGCCTCCATCTCAACTGGCAGCACCTGTTCAGTGCATAAATGCCAGTCGATGGTCTTTAATTATGCAAAAAAGCCCGTCCGATTCGAGAGGCAAAACAGCCTCGATGCTCTATCATTCAGACCGGGCATCTTTAAGAGTATTCGTAGGGAGAAGCCAACCGAACAGGCGAATCCAGAACCACAGCAGACAAAGAATATCAAAAAGTCCTCACAATTCCTTGCCGAGAGCAAGCACGTTTCTCTGCCATCGGATAGCGAAGATGAGTTGGAACTTAAAGCCCAAAAACGTTATTTGCAAGATCAGTTTGTGAAAAAGCTCAATAGCAGTAAGAATGATGAGAAGACTGGCACAGACGCAACGTCAAAAATCTCGGAAATTCCAAGACCAAATCCAGAAAACGAGCAACTAAACACTGTATGCGAAAAGTATGAGCAGGAAACGAGTGATCTGCGATCCAATCTTAATTTGATCGACAAACTAGAATTACAGCTACAAAACTTACAGATTGATAAGGAAAAGCTGACCAACAAGTTGTCACGCGTGGAGTCAATATTAAACAAACACGATTTAAATCCAGAAAAAGCTGTTCTAAAATTAAGAAAATTGCTATTGAACCAAGCACAAACGGCAAGAGTGCATCCGCGTAAAGCGGATACACTTATTGATGAAGATGAATTAACCAAGAAAGAAGAATTCAAGTCTTGCACGGAATTTTCTCGTCAGGAAGACGTTAAGTTGACGCCAACTCAAATAAAAAATCAAAAATCAAATACTAGGTGTTCGCAAACTAAGGTCAGAAAGAGCCTAAAGGTTCGAGATCTGCGGC
CL344	CGAGAGGTACCATGAACACCCCGGGAATTAACCTGATCAAGGAGGACATGCGCGTGAAGGTGCACATGGAGGGCAACGTGAACGGCCACGCCTTCGTGATCGAGGGCGAGGGCAAGGGCAAGCCCTACGAGGGCACCCAGACCGCCAACCTGACCGTGAAGGAGGGCGCCCCCCTGCCCTTCAGCTACGACATCCTGACCACCGCCGTGCACTACGGCAACCGGGTGTTCACCAAGTACCCCGAGGACATCCCCGACTACTTCAAGCAGAGCTTCCCCGAGGGCTACAGCTGGGAGCGCACCATGACCTTCGAGGACAAGGGCATCTGCACCATCCGCAGCGACATCAGCCTGGAGGGCGACTGCTTCTTCCAGAACGTGCGCTTCAAGGGCACCAACTTCCCCCCCAACGGCCCCGTGATGCAGAAGAAAACCCTGAAGTGGGAGCCCAGCACCGAGAAGCTGCACGTGCGCGACGGCCTGCTGGTGGGCAACATCAACATGGCCCTGCTGCTGGAGGGCGGCGGCCACTACCTGTGCGACTTCAAGACCACCTACAAGGCCAAGAAGGTGGTGCAGCTGCCCGACGCCCACTTCGTGGACCACCGCATCGAGATCCTGGGCAACGACAGCGACTACAACAAGGTGAAGCTGTACGAGCACGCCGTGGCCCGCTACAGCCCCCTGCCCAGCCAGGTGTGGTAATATCTAGAGGCAG
CL349	GGCAAGATCTCAAAATGGCACTTTCCGCAACGAAGG

CL350	GTACACTAGTCAAGCGGCCGCGAGGAATTCGAGGTTAACGAGGAGCTCATAAGCAAACCGGATCTAACCTAGG
CL351	CTAGCCTAGGTTAGATCCGGTTTGCTTATGAGCTCCTCGTTAACCTCGAATTCCTCGCGGCCGCTTGACTAGT
CL381	GACCAGATCTCACCACCAACATGGCACTTTCCGCAACGAAGGACACTAGTCCATGGACCAACATGGTGAAGAGGGTAACAGCGAGCAAATACGATGTAGTCTCGGTCAAGACGCTGCTGATGTTTATCAACAGCAAACTGGATTGCGAATTGCGAGGCTTTGAGGATCTGAAAACAGGAGCAGTATATTGCCAACTGATGCACAGACTCTTTCCCAATTCGATACAAATTCATAAGGTCAAGTTTTATACGAACGACATCAGCGACTTTCAGTTAAATTTCCGGTTACTAAACAATTGCTTTCAAAAACTAAGAGTAACCGTTTATATGCCAGTGCATGAGTTAACTCTGGGACACAATCAAGTGGTCTTCTGCAATTGGATATACAAGTTCTATGAAGCAAACGACAAGGGGAACGAATACGATGCCAGAAAGGTGAGAAAAGGCTCGCCAATCGGTTTAGACAATAGTGCGGTAAAGTCACTGCCCGGCACGGCGGCAAGCGGCGTGTCCAGCAGCTATCGACGTGGCCCATCGGCAACGACACGCCCAGCAATGACGTCTGCAGTGAAGCCCACAGTATCCAAGGTGCTGCCGCGCACGAACAACGCAGCCCCAGCGAGCAGAATAAACGCCTGTGCCAACAGCACGGGCACGGTCAAGAAGAACGACGTGAGCAATTCGGTCAACAATCAACAAATAGAAGAGATGTCAAATCAGGTGATGGATATGCGCATAAACCTGGAGGGATTGGAAAAGGAGCGAGACTTTTACTTCTCTAAGTTGCGGGATATTGAAATTCTTTGCCAAGAAGCCGATGACGCCGAGGCGCATCCGATCATACAAAAGATTTTGGACATCTTATATGCGACTGAGGATGGTTTTGCGCCGCCTGACGATGCACCACCAGAGGACGAGGAGTATAGCGGCCGCATCCTTG
CL385	GATCGCGGCCGCCTTGTACAGCTCGTCCATGCC
CL396	AAAGAGCTCTAGAGTTTGATGCAAATGTTGCGTCC
CL397	GGAAGGATCCGGATCAGAGGCAATGGAAGGC
CL405	ATGTGGGATCCGATGTCAAGCAGGTCCACGAG
CL406	ACAGCGAATTCTTTGATATCGCGCTACAATATAC
LP011	CATCATTCTTACTGCTATTGAG
LP017	GCCAGCGCGCCACCTTAGCTTGCACATACG
LP018	GCCAAGATCTCGAACCTTTAGGCTCTTTCTGACC
LP021	CAGAATCGGTAAGAATAGCTG
LP023	GCGGATTCCCAATCGCCAGCTAGCCAAGAAGCACCGAAGTTGGC
LP024	GCGATGCCCGCAGCTGTTGCTAGCACTACCTCAATTGGATCCAG
LP025	CCTAATCCGGACCCACCAGCTAGCGCAGCTAAGTCACTGTTTGG
LP026	AAGACACAGGTGGCTAAGGCTAGCGCCGAAAAACAAAAGGAACA
LP027	GATGATTTCTATTCCCATGCTAGCTTCTCTGCCAATCGATCACG
LP028	GGCCGCAATGTTTACAACGCGAAAAG
LP029	CTAGCTTTTCGCGTTGTAAACATTGC
LP030	CTAGCTAAGCAAACCGGATCTAAC

LP031	CTAGGTTAGATCCGGTTTGCTTAG
LP037	GCTCACCATGCCGTCGACACTATTGTCTAAACCGAT
LP038	GACAATAGTGTCGACGGCATGGTGAGCAAGGGCGAG
LP039	GGACACTAGTTATAAGGTAGCCTCCATCTC
LP043	GAATGTACCTCAGTCAACACAG
LP044	CCTTAGTTTGCGAACACCTAG
LP045	TAATACGACTCACTATAGGGAGAGAAGCTGAGCAAAACCAAAG
LP046	TAATACGACTCACTATAGGGAGATTCACGGTTTCCTTCGACT
LP049	CCGAACGAAGACCCTGCAGACTCCAGTTATATTAACTTGTTTTAGAGCTAGAAATAGCAAGTTAAAATAAGGCTAGTCCGTTATCAACTTGAAAAAGTGGCACCGAGTCGGTGCTAACAAAGCACCAGTGGTCTAGTGGTAGAATAGTACCCTGCCACGGTACAGACCCGGGTTCGATTCCCGGCTGGTGCAGCTATTGAACCAAGCACAAAGTTTTAGTCTTCGGTTCG (gRNA1, gRNA2)
LP050	CCGAACGAAGACCCTGCAGATGTTTATCAACAGCAAACGTTTTAGAGCTAGAAATAGCAAGTTAAAATAAGGCTAGTCCGTTATCAACTTGAAAAAGTGGCACCGAGTCGGTGCTAACAAAGCACCAGTGGTCTAGTGGTAGAATAGTACCCTGCCACGGTACAGACCCGGGTTCGATTCCCGGCTGGTGCAGATGCTCTATCATTCAGACCGTTTTAGTCTTCGGTTCG (gRNA1, gRNA2)
LP054	CGCCAGCTAGCCAAGAAGCACCGAAGTTGGCCTTCGGTGGAATTGCCGCACCTGTGTTCGGAGACGCCAATCCTTTTGGAGGCCACAAAGTTAATTTGCAAAAATCAGATGGCAAGGAAGAACCAAAATCTATCATTGGCGGAACCCCATTGTTATTTGGAGGGTCCAACGCCTTTGGAATACCCAAAATTGAAACCCAGTCACCTGCTAAAGACTTTGTGTTTGGCAGTGCACCCGCTTTTGGACAAATGGCAACCTTCTCATTTACAGCTGCAAAAAATGAAAAGGAAAAGGACATAACCTCGAATAACACCACTGATCTTAAAGCCGAAGGCAAGGAAAAGAAGGAGTTGGTGCCGGAGACGACCAGCACATTTGCCGATTTGGCCAAGACAGGCAGCACGTTTGCAGATTTGGCAAGCAATCCGGGCTAAGCAAACCGGATCTAACCTAGGCTAGTT
LP055	CGCCAGCTAGCCAAGAAGCACCGAAGTTGGCCTTCGGTGGAATTGCCGCACCTGTGTTCGGAGACGCCAATCCTTTTGGAGGCCACAAAGTTAATTTGCAAAAATCAGATGGCAAGGAAGAACCAAAATCTATCATTGGCGGAACCCCATTGTTATTTGGAGGGTCCAACGCCTTTGGAATACCCAAAATTGAAACCCAGTCACCTGCTAAAGACTTTGTGTTTGGCAGTGCACCCGCTTTTGGACAAATGGCAACCTTCTCATTTACAGCTGCAAAAAATGAAAAGGAAAAGGACATAACCTCGAATAACACCACTGATCTTAAAGCCGAAGGCAAGGAAAAGAAGGAGTTGGTGCCGGAGACGACCAGCACATTTGCCGATTTGGCCAAGACAGGCAGCACGTTTGCAGATTTGGCAAGCAATCCGGGCGGCACTTTTGCGGACTTAGCGAACAAGACGGGCAACGACTTTGCCAACCTGTCGGCCAATAGCCAGGGCACTACCGTGGGATTCAACAAGTCCGCCGGGGGCGGCTTTTATAACCTCACGCATCAGAACGCTTTCAAGAACTTCGAGTCGCCGCAAGCCACATAAGCAAACCGGATCTAACCTAGGCTAGTT
LP056	CGCCAGCTAGCCAAGAAGCACCGAAGTTGGCCTTCGGTGGAATTGCCGCACCTGTGTTCGGAGACGCCAATCCTTTTGGAGGCCACAAAGTTAATTTGCAAAAATCAGATGGCAAGGAAGAACCAAAATCTATCATTGGCGGAACCCCATTGTTATTTGGAGGGTCCAACGCCTTTGGAATACCCAAAATTGAAACCCAGTCACCTGCTAAAGACTTTGTGTTTGGCAGTGCACCCGCTTTTGGACAAATGGCAACCTTCTCATTTACAGCTGCAAAAAATGAAAAGGAAAAGGACATAACCTCGAATAACACCACTGATCTTAAAGCCGAAGGCAAGGAAAAGAAGGAGTTGGTGCCGGAGACGACCAGCACATTTGCCGATTTGGCCAAGACAGGCAGCACGTTTGCAGATTTGGCAAGCAATCCGGGCGGCACTTTTGCGG

	ACTTAGCGAACAAGACGGGCAACGACTTTGCCAACCTGTCGGCCAATAGCCAGGGCACTACCG<br>TGGGATTCAACAAGTCCGCCGGGGGCGGCTTTTATAACCTCACGCATCAGAACGCTTTCAAGAA<br>CTTCGAGTCGCCGCAAGCCACAGAAGAGTGCGATGATGACGGCGACGCGACCACTGATGACA<br>ACTACGATCCGCACTATGATGCCATTGTAGAGCTGCCGGATGAGATAGTTGTCACCACGGGAG<br>AGGAGAACGAGACTAAGCTGTTTGGCGAGCGGGCGAAGCTTTATCGCTATGACGCCGAATCGA<br>AACAATGGAAGGAGCGAGGTGTCGGTGAGATAAAGGTGCTGGAGCACCCGGAGCTGCAGAC<br>ATTCCGACTGATCATGCGGCAGGAGCAGATCCACAAGCTGGTGCTTAACATGAATATCTCCGCC<br>TCCCTGCAAATGGATTACATGAACGCGCAGATGAAGAGCTTCCTGTGGGCCGGCTACAACTAC<br>GCGGTGGACGCAGAGGGTAAAGTTGACACCGAGGGCGTCCTGGAACGCCTTGCCTGTCGATTC<br>GCCAAGGAAGAGATCGCCAGTGAGTTCCTCAACACGGTCAATTCGTGCATAAAACGAGCCAAG<br>TAAGCAAACCGGATCTAACCTAGGCTAGTT
RAS42	CGAATTCATGGTGAGCAAGGGCGAG
