# Supplementary material for: Nuclear elongation during spermiogenesis depends on physical linkage of nuclear pore complexes to bundled microtubules by Drosophila Mst27D
Source: PLoS Genet. 2023 Jul 10;19(7):e1010837. doi: 10.1371/journal.pgen.1010837 (PMC10359004; doi:10.1371/journal.pgen.1010837)
Supplement: Attachment — Submitted filename: Response to Reviewers.pdf [file pgen.1010837.s025.pdf]

Response to Reviewer #1:

We thank reviewer 1 for the overall positive appreciation of our original manuscript as well as for insightful and very helpful comments. Our response to these comments is:

1. Figure 1: The authors present the affinity pulldown with Mst27D::EGFP where they identify Nup358 and also its binding partner RanGAP. What about CG2233 ?? The authors should at least mention or discuss this apparently specific hit.

As suggested, we have inserted an additional paragraph commenting on CG2233 (lines 156-163 in the revised version).

2a. Figure3A: why does mcherry::Nup358 spread to MTs with FL-Mst27D but not with CT-Mst27D that actually binds Nup358 (Figure 2) and by itself also localizes to MTs around the nucleus (3A, middle panel) ?

While Mst27D_CT-EGFP is indeed not present exclusively at the NE, the data shown in Fig 3A (middle panel) should not be viewed as an unambiguous demonstration that Mst27D_CT-EGFP "localizes to MTs around the nucleus". The evident absence of a perfect spatial correlation between the perinuclear Mst27D_CT-EGFP signals and anti-α-Tubulin indicates that at least a considerable fraction of the perinuclear Mst27D_CT-EGFP is associated with cellular structures (ER?) other than MTs. It is possible, though, that some Mst27D_CT-EGFP might be associated with perinuclear MTs. Mst27D_CT-EGFP expressing S2R+ cells clearly display some EGFP signals associated with moving comets, as already stated in the original manuscript. These comet signals, which are particularly evident in time lapse movies, are weaker but otherwise highly similar to those observed with Eb1-EGFP. Although heterodimerization of Mst27D and Eb1 was not detected in our co-IP experiments, Mst27D_CT might be able to heterodimerize with Eb1 or with the uncharacterized Eb1 family protein encoded by *CG18190*, which is expressed in S2R+ cells according to FlyBase RNA-Seq data. We speculate that such heterodimerization might explain the comet signals Mst27D_CT-EGFP expressing S2R+ cells. Potentially, low amounts of such heterodimers might also bind along MTs, as known for Eb1 and contribute to the EGFP signals in the perinuclear region of Mst27D_CT-EGFP expressing S2R+ cells, in which also MTs are abundant. However, this putative MT binding of Mst27D_CT is far less efficient than that of full length Mst27D. Particularly clear support for this conclusion derives from analysis of Mst27D-mCherry and Mst27D_CT-mCherry in spermatocytes during progression through M I. The former displays a striking spindle association (S6A Fig), while the latter is on the spindle envelope (S9A Fig). To improve the documentation of the subcellular localization of Mst27D_CT-EGFP in S2R+ cells in our manuscript, an additional column was added into the revised Fig 3C, displaying still frames after time lapse imaging of representative cells in interphase and mitosis, respectively, for comparison of subcellular localization with other EGFP-tagged Mst27D variants. Text referring to this addition was added into the result section (lines 234-240 in the revised version).

Although Mst27D_CT-EGFP does not bind MTs as efficiently and in the same manner as full length Mst27D-EGFP, it is certainly true that the former is not present exclusively on the NE, and we agree with reviewer 1 that the image presented in Fig3A, middle panel, can generate an impression that Nup358-mCherry appears to be recruited less efficiently to the non-NE fraction of Mst27D_CT-EGFP, compared to the Nup358-mCherry recruitment to MTs that is induced by Mst27D-EGFP. However, we would like to point out some technical limitations that preclude clearcut conclusions concerning different mCherry-Nup358 recruitment efficiencies of Mst27D-EGFP and Mst27D_CT-EGFP based on our current analyses. There is considerable cell-to-cell variation in expression levels and ratio of the EGFP- and mCherry-tagged proteins after S2R+ cell transfection, precluding simple quantification of recruitment efficiencies. Moreover, although mCherry-Nup358 is predominantly localized at the NE and in NE-associated dots (annulate lamellae?), weak signals can also be detected at other locations (including possibly ER and/or perinuclear MTs) in strongly expressing cells after single transfection without an EGFP expression construct. These non-NE signals further complicate a precise

quantification of recruitment efficiencies. Non-NE localization of Nup358 has also been reported in human cells (for example Joseph and Dasso, 2008).

Assuming that mCherry-Nup358 is indeed recruited less efficiently to the fraction of non-NE-associated Mst27D_CT-EGFP compared to the Nup358-mCherry recruitment to MTs induced by Mst27D-EGFP, what might explain the difference in recruitment efficiency? We speculate that the putative heterodimers of Mst27D_CT-EGFP with Eb1 or *CG18190* protein, which might explain the non-NE localization of Mst27D_CT-EGFP, might have a reduced Nup358 binding affinity.

2b. How do Mst27D::GFP and cherry::Nup358 localize without co-transfecting the other protein? The authors should include this control.

Images illustrating the localization of Mst27D-EGFP and mCherry-Nup358 in S2R+ cells without additional expression constructs are displayed in both the original and the revised version (Fig 3C: Mst27D-EGFP; S1 Fig: mCherry-Nup358).

2c. The authors state that MT binding of Mst27D is mediated by its CH domain and homodimerization by its CC in the C terminus. Why? However in Figure 3A they show that Mst27D_C that has the CC but lacks the Calponin homology domain localizes to MTs. Why? The more clearcut answer to this question would be to transfect a Mst27D construct that just lacks the CC domain.

The statement that "MT binding of Mst27D is mediated by its CH domain" is based primarily on the evidence that expression of the CH domain of Mst27D as a chimera with EB1's CT part (which does not bind to MTs but confers dimerization) results in highly efficient co-localization with MTs.

A statement that homodimerization of Mst27D would be mediated "by its CC in the C terminus" was not made anywhere in both the original and the revised version. As clearly supported by our evidence from co-IP experiments (Fig 3B), we state that the CT region mediates self-association ("Thus, Mst27D appears to form homodimers mediated by the CT region"; in the revised version line 217).

For our response to the question of why Mst27D_C that has the CC but lacks the Calponin homology domain localizes to MTs, see our response to comment 2a above.

We agree that additional analyses with Mst27D variants lacking the CC region are likely to provide additional insight into the functional domains of Mst27D but consider these to be beyond the scope of our present study that does not state any conclusions that depend on such experiments.

3a. line 244: The authors write that their transgenes rescue mutants of *mst27D* or *nup358*. Which mutants were used (references !) ? What are the rescued phenotypes? Lethality or male sterility in the case of *mst27D* (where I suppose they use the mutant generated in this study)?

The information answering these questions was given further below in the original manuscript. To alert readers to these subsequent additional explanations, we have placed "As described further below, …" at the start of the sentence first mentioning that the transgenes rescue the mutants (line 264 in the revised manuscript).

3b. line 248: it would be helpful to relate the "S5", "S6" staging as referred to in the results section (Figure 4, S4) to the initial description of sperm maturation, for example by explaining the "S" stages in Figure 1 or by text in the introduction.

As suggested, we have added text into the introduction, giving a brief explanation for the spermatocyte maturation stages, as well as the reference (Cenci et al. 1994, J Cell Sci) that provides the detailed description of the stages (lines 96-100 in the revised version).

3c. Expression of the Mst27D and Nup358 transgenes in spermatocytes: Could the authors show here that Mst27D localizes to the NE hemisphere that contains NPCs. Actually the authors do show this very nicely in Figure S6A where they present stills of a movie during NE polarization. I would suggest to move this particular display item (S6A, lower panel) into the main Figures (Fig 4) which

would further illustrate their conclusion that Mst27D associates with NPCs (and is thus devoid of non NPC containing NE)

Fig 4 illustrates the subcellular localization of Mst27D-mCherry in spermatocytes, in which the NPCs are not yet restricted to a hemisphere. The confinement of Mst27D-mCherry to the NPC-NE occurs only later in spermatids, and this is shown in Fig 5 (and illustrated further in Fig S6A and S1 Movie).

4a. Figure S4: the dendra::Mst27D should only spread within the NPC containing hemisphere. Can the authors comment on this?

The experiments addressing the dynamics of Mst27D association with the NE by photoconversion of Mst27D-Dendra2 in a restricted region of the NE were done in in S6 spermatocytes, in which the NPCs are still distributed throughout the NE (before confinement to a hemisphere in spermatids). Analogous analyses in spermatids are not feasible technically with our standard microscope methods because of the far smaller size of spermatid nuclei.

4b. the authors interpret their data on Dendra::Mst27D distribution after photoconversion as "intermittent episodes of transient association" with NPCs (line 263), because NPCs are believed to be immobile within the NE. Referring to this restraint, which was however discovered in somatic cells: how is eventually NPC distribution within the NE during spermatogenesis- which is very nicely demonstrated in Movie S1-achieved? Mobile NPCs, for example due to an altered lamina composition compared to somatic cells would not only allow NPC redistribution but also offer an alternative interpretation of Mst27D dynamics.

Previous analyses of known components of the nuclear lamina (including lamins; Fabbretti et al. 2016. PLOS One. Confocal Analysis of Nuclear Lamina Behavior during Male Meiosis and Spermatogenesis in Drosophila melanogaster) have not provided evidence for a change in lamina composition in late spermatocytes that might explain our observations concerning Mst27D-Dendra2 mobility.

The NPC re-distribution from spherical to hemispherical in early round spermatids (as analyzed by time lapse imaging here for the first time; S1 Movie) is certainly a fascinating interesting process, but this process (designated as NE polarization in our manuscript) is not understood currently at a mechanistic level. Our findings described in this manuscript indicate that NE polarization does not depend on dynamic MTs and that Mst27D re-distribution is not a down-stream consequence of Spag4 (and hence Dynein/Dynactin) redistribution. Anti-laminDm0 signals (Fabbretti et al. 2016) indicate that lamins undergo a redistribution during NE polarization that corresponds to that of Mst27D (and Spag4 and Dynein/Dynactin). Causes and functional significance of lamin protein redistribution are unknown.

5. There is a major confusion with the referring of results, main Figures and legends with respect to Figures 4, 5 and 6. The text in the results section (starting from line 265) describes the strategy to knockdown by the degrade system. The respective data is presented in Figure 4B, C but the results section says Figure 5A and later 5B,C (line296). However Figure 5 presents results about nuclear elongation. Later (from line 316) the text talks about results on nuclear elongation and NE shedding that are in fact presented in Figure 5A, yet the results section refers to Figure 6 (lines 319, 332).

We are sorry for having overlooked these awkward mistakes during proofreading of the original version; they are corrected in the revised manuscript.

6a. The authors present data obtained with peptide antibodies that they have raised to detect endogenous Mst27D. The antibody is specific, because the signal is gone in "Mst27D-" tissues (Figure 5D). The legend to this Figure (line 1602) says "preparations of testes with or without endogenous Mst27D expression. This phrasing is ambiguous. The authors should state that it derives from the 0 mutant that they have generated which is presented in Figure 6.

As suggested, we have changed the text of the figure legend (lines 1664-1668) in the revised version). The precise genotypes analyzed are also listed in S3 Table that was provided already with the original version.

6b. With respect to the same figure (5D) I do not see which display item should support the statement made in line 345/346 that “a specific Mst27D signal was not observed before the onset of nuclear elongation”. I see only specific signal in all three panels (also in “early”), which is gone in Mst27D- tissue.

We have corrected the corresponding statement so that is now in accord with the presented figure (line 367 in the revised version).

7a. Analysis of mst27D mutants: The authors state that based on their analysis of mst27D mutants, the protein is required for nuclear elongation. This is well supported by the displayed stills of a movie from mst27D mutant spermatids expressing GFP::Nup358, compared to control spermatids (Figure 6E). However in the panels below (6F and 6G) nuclei in mutant spermatids appear also long, yet their shape is aberrant. The same holds true for Figure S9 or S12 B where, in later stages, nuclei seem to have well elongated despite the lack of Mst27D. Since the authors make a strong point in revealing the essential role of Mst27D for nuclear elongation they have to discuss or explain this discrepancy.

This discrepancy is also highlighted by the following sentence (line 564) where the authors discuss the independent roles of Spag4 and Mst27D: “ This preferential localization of Spag4-EGFP on the basal body increased further **during nuclear elongation in both controls and *mst27D* mutants** (S12B).”

As already clearly reported in the original manuscript, the extent of morphological abnormalities that develop during the stages of nuclear elongation in Mst27D mutants varies from cyst to cyst. In some of the mutant cysts, nuclear elongation clearly occurs to a considerable extent. In the discussion, for example, we stated: “A contribution to nuclear elongation by players other than Mst27D is also suggested by the residual elongation that can be observed in *Mst27D* null mutants” (line 686) followed by a discussion of potential explanations for this residual nuclear elongation. To assess the frequency of nuclear elongation defects in the *Mst27D* mutants despite their variability, we have scored all the spermatid cysts that were positive for Tpl94D-mRFP in control and *Mst27D* null mutant cysts. The transition protein Tpl94D-mRFP is expressed only very transiently during the late phase of nuclear elongation in controls. As our evidence had not revealed indications for an altered timing of Tpl94D-mRFP accumulation in *Mst27D* null mutants, Tpl94D-mRFP should be an excellent marker to score the morphological appearance of spermatid nuclei during comparable stages in control and *Mst27D* null mutants. As reported in the original and revised version, 98% of the Tpl94D-mRFP positive cysts displayed abnormal nuclear shapes in *Mst27D* mutants (n = 197 cysts from 10 testes), while only 1.4% of the Tpl94D-mRFP positive cysts contained nuclei with irregular non-canoe shapes in controls (n = 191 cysts from 10 testes) (line 456 in the revised version). Based on this (and a lot of additional evidence), we have concluded that Mst27D is required for normal nuclear elongation. We have been careful in writing “normal” and have avoided stronger statements like “the protein is required for nuclear elongation”. Checking once more, we have made sure that our phrasing is appropriate and have corrected the one occurrence overlooked in the original version (line 383 in the revised version). Moreover, to avoid misunderstanding, we now report earlier and more explicitly that residual nuclear elongation occurs to a variable extent in the *Mst27D* null mutants (lines 433-435 in the revised version). In addition, the misleading sentence indicated by reviewer 1 (starting in line 564 of the original manuscript) was changed to: “This preferential localization of Spag4-EGFP on the basal body increased further during nuclear elongation in controls and during the corresponding stages also in *Mst27D* mutants (S12B Fig).” (lines 590-591 in the revised version)

7b. In Figure S9 the authors rescue or do not rescue the nuclear elongation phenotype by expressing transgenes for versions of Mst27D. The figure should also contain a panel with just the mutant without any transgene to better assess the original phenotype.

The results illustrated in S9 Fig are presented to support our conclusion that nuclear elongation was restored back to normal in *Mst27D* mutants expressing *g-Mst27D-mCherry*, while *g-Mst27D_CH-mCherry* and *g-Mst27D_CT-mCherry* did not result in normal nuclear elongation. This figure and our text do not intend to address whether expression of either the CH or the CT part of Mst27D protein in the mutants influences the phenotype. While this does not seem to be the case, subtle differences could not be resolved by adding single images from the mutants into the figure because of the phenotypic variabilities (see above).

8. Figure 7: The authors should indicate the respective labels for the fluorescent proteins in panels B, C and D.

Fig 7 of both the original and revised version has these labels actually on top of the panels B-D.

9. MT dynamics and bundling. While the name decolcemid is used in the text, the drug's other name colcemid is used in the Figure legend (S10). To avoid confusion the authors should stick to one terminology.

As suggested, only one designation is now used throughout the revised version: "demecolcine" (The designation "decolcemid" was not used anywhere in the original version).

10. Minor problems:
- Line 146: the authors talk about Nup358 and its embedding in the NPC. They mention the NPCs outer ring, but non-experts won't understand. A brief explanation of NPC architecture in the introduction would help, otherwise be less specific
- Line 267: " …that is assembled into **a** GFP-specific ubiquitin ligase"
- Line 279: "For analysis we used testes dissected from larvae at the early pupal stages". This is confusing, testes were from larvae or from pupae?
- Line 409: … "nuclear elongat**ion** was limited…
- Line 425: was also supported **by** the conclusion (for example)

Thanks for pointing out these mistakes, which we have corrected.

Response to Reviewer #2:

We thank reviewer 2 for the evaluation of our manuscript, which was considered to be comprehensive and a very elegant piece of work. While no major criticism was forwarded, we address below the following comments: "However, I was left a bit unsatisfied about the lack of statistical support about the degree of homology with EB1 (Figure 1 alignment) and the relative history of Mst27D. A previous paper suggested that this gene was a result of retroposition of CG15306, so it would have been useful to see at least some acknowledgement of the evolutionary relationships and possible retention of these genes in different Drosophila species, which should be very straightforward to do. The authors comment on how these additional EB1 homology genes might be slightly redundant for nuclear elongation function with Mst27D- it would have been really cool to see if double mutants of Mst27D and CG15306 are completely sterile due to redundancies in nuclear elongation function (whereas spag4 mutants are sterile). However, I do concede that is beyond the scope of the current paper (although the evolutionary description would be still nice here) … "

We fully agree that the evolutionary history of *Mst27D* and the other Eb1 family genes, as well as the extent of functional redundancies among these genes, is of great interest and deserves further study. However, we indeed consider such analyses, for which our competence is also partly rather limited, to be beyond the scope of this present paper. To respond to the comments of reviewer 2 in the context of our paper, we have changed our manuscript as follows:

1. Concerning the degree of homology with EB1 (Figure 1 alignment), we now describe in detail how the alignment was generated in Materials and Methods (lines 1049-1053 in the revised version).

2. Concerning evolutionary history and potential functional redundancies between *Mst27D* and additional EB1 family members, we have extended our corresponding comments in the Discussion (lines 716-722 in the revised version), including the finding that *Mst27D* appears to have arisen from *CG13506* and the corresponding reference (Pan and Zhang 2009).

Response to Reviewer #3:
We thank reviewer 3 for the careful evaluation of our original manuscript. Beyond a very positive overall assessment, minor comments to improve some aspects of the manuscript were provided. We respond to these insightful and very helpful comments as follows:

General comments:
1. The section of the Fig. 1B legend that describes relocalization of the NPC "into a part of the NE that covers only a hemisphere of the spherical nucleus in early spermatids" does not entirely reflect the relevant biology. Restriction of NPCs to a hemisphere of the spherical early spermatid nucleus actually occurs prior to the stage shown in the left-most diagram in 1B, and the basal body attaches in the middle of the hemisphere where the NPCs are located (see Galletta et al. 2020). After this, there is a 90°shift in the distribution of NPCs such that their distribution resembles what is shown in the left-most diagram. It might help to clarify this by mentioning the earlier step and saying it is not shown. This would avoid confusion on the part of readers who are familiar with the earlier step.

Fig 1B reproduces the scheme from the classic review by Lindley and Tokuyasu (1980), and indeed, we were also confused initially by the absence of the earlier stages of NE polarization in this scheme. Thus, we entirely agree that a clarification should be helpful. We have inserted such a clarification into the discussion (lines 627-632 in the revised version), where it is even better positioned than in the legend of Fig 1B.

2. Three experiments that were performed using S2R+ cells could be extended to show whether the results are physiologically relevant in vivo:
a) To confirm the interaction of Mst27D and Nup358, it would be nice to show coIP of the two proteins from testis extracts.

The interaction of Mst27D-EGFP and Nup358 was clearly observed in our AP-MS experiments that were done with testes extracts (four replicates; Fig 1D). Thus, we think that the physiological relevance of the Mst27D-Nup358 interaction would obtain at most marginal additional support by evidence from the suggested analyses of co-immunoprecipitation by western blotting instead of MS.

b) It appears that mCherry-Nup358 localization at the NE is patchy when coexpressed with Mst27D_CH_EGFP in S2R+ cells. It would be nice to know whether expression of Mst27D_CH_EGFP also interferes with proper NE distribution of Nup358 in spermatocytes.

The patchiness of mCherry-Nup358 localization varies somewhat from cell-to-cell in transfected S2R+ cells and in a comparable manner without and with co-transfection of constructs for co-expression of the distinct Mst27D-EGFP variants.

c) Given the dramatic bundling of MTs caused by Mst27D overexpression in S2R+ cells, it would be interesting to know whether this also occurs upon Mst27D overexpression in the male germline.

We fully agree that further analyses in spermatids for clarification of the physiological relevance of the MT bundling activity of Mst27D observed in S2R+ cells are of considerable interest. However, we consider such experiments which require the generation of additional transgenic strains to be beyond the scope of our present manuscript.

3. The levels of Mst27D protein appear reduced following Nup358 knockdown (S2R+ cells) or degradation (testes). It would be helpful to include immunoblots showing the extent of this effect, especially in the testis degradFP experiments.

We agree that the suggested analyses by immunoblotting should result in additional information concerning the extent of reduction of the Mst27D protein levels in response to Nup358 RNAi and deGradFP. However, such additional information would not provide further help in resolving the crucial question (Does Mst27D localization at the NE depend on Nup358?) that motivated these experiments.

4. The authors state that Mst27D mutants have "partial individualization defects". To support this claim, it would be helpful to include low-mag images of whole mount testes stained with fluorescent phalloidin, as this would indicate to what extent individualization complexes progress along the spermatid bundles in Mst27D mutants vs. controls.

We have not taken up this suggestion, as we consider such low-mag images to be of very limited help given the phenotypic variabilities observed in *Mst27D* mutants.

5. Spag4 does not appear to be tightly juxtaposed to the nuclear envelope in the early elongating stage spermatids shown in S12B. Could a defect in basal body attachment precede the defects in nuclear elongation in Mst27D mutants?

We have not observed an obvious defect.

6. The results section describing the difference in expression of transgenes with SV40 vs. endogenous 3' regulatory sequences is rather long and could be shortened.

As suggested, the corresponding paragraph was made more concise (lines 368-378 in the revised version).

7. The student t-test requires that data be normally distributed. Given the distribution of datapoints in the graphs in Fig. 4D, S3C, and S8B, a Wilcoxon rank sum test might be more appropriate.

As suggested, we have reanalyzed the data displayed in these figures using the Wilcoxon rank sum test (line 1277 in the revised version). The results of these statistical tests have not changed the original conclusions.

Comments on the figures and figure legends:

Fig. 3A: It would help to show the green and red single-channel images in grayscale and to change the red to magenta in the merged image to help colorblind readers.

Fig 3A displays three different channels in single channel images (green for Mst27D-EGFP variants, red for mCherry-Nup358 and grey values for anti-αTub) as well as merged images. The suggested change of red to magenta would result in increased difficulties for the assessment of co-localization of co-localized red and green signals with anti-αTub signals. This drawback led us to ignore the suggestion. As all channels in Fig 3A are displayed not just in a merged image but also as single channel images, it should still be accessible also to colorblind readers.

Fig. 6C: The absence of a label under the right-most set of datapoints (shown as red dots) is confusing. It would help to indicate that these lack a transgene.

Fig 6C was changed as suggested.

S7 Fig: The images in panel C appear to be 3D renderings, or at least qualitatively different from the images in the other panels. If true, this should be described in the legend.

As now stated in the revised legend of S7C Fig, the original images acquired with spinning disk confocal microscopy were deconvolved before maximum intensity projection for this panel.

S9 Fig: It would help to include labels indicating that the transgenes in panel A are expressed in a Mst28D+ background and those in panels B-D are in a Mst27D- background.

S9 Fig was changed as suggested.

S10 Fig: It would help to include a label in panel B indicating that the sample was treated with colchicine.

S10 Fig was changed as suggested.

Minor corrections:
line 62: pluralize “Histones”
line 73: change “role” to “roles”
line 74: unclear what is meant by “in context with”; perhaps “in the context of”?
line 91: missing “a” before “limited”
lines 102-103: for clarity, suggest moving “and the NPC-NE” (line 102) after “shaped nucleus” (line 103)
line 119: missing parenthesis after references
line 133: the abbreviation “g-“ is used throughout to indicate genomic upstream sequences, but this is never defined; it would help to define this here”
line 154: refer to Fig. 2A and 2B after “N terminus”
lines 155, 156, 160: replace “2B” with “2C” (three instances)
line 158: should this be “aa 151-424”, rather than “aa 141-424”, as suggested in Fig. 2A?
line 174: replace “2C” with “2B”
line 179: fix typo in “indicated”
line 186: delete “in” before figure callout
line 189: replace “highlighted” with “highlight”
lines 229-230: the results described here (line 229) are not present in S2 Fig (line 230)
lines 237-238: replace “S4 Fig” with “S3 Fig” (two instances)
line 267: missing “a” after “into”
line 272: the promoter “exumP” does not appear to be described anywhere in the text; please describe its origin, including what gene it is from, here
line 278: replace “5A” with “4B”
line 289: replace “5C” with “4D”
lines 290, 292,296: replace “5B,C” with “4C,D” (three instances)
lines 319, 332, 334: replace “Fig 6” with “Fig 5” (three instances)
line 383: replace “were” with “we”
line 392: replace “codes for” with “encodes”
line 409: replace “elongated” with “elongation”
line 463: delete “from”
lines 600-601: for clarity, suggest changing “appeared conceivable at first” to “initially appeared conceivable”
line 619: delete comma after “mutants”
lines 641, 650: awkward to use “according to our proposal” twice in two paragraphs; perhaps replace the first instance with “our proposed model” or “our proposed mechanism” and the second with “We propose that”
line 654: replace “be” with “by”
lines 713-718: paragraph is awkward as written; suggest moving “The MT bundles…[25,26].” (lines 717-718) before “While a single MT bundle…” (line 713)
line 747: replace “does” with “do”
line 780: replace “present” with “presence”
line 808: clarify whether the mutation described (N92T) is in the Mst27D coding region
lines 909-910: replace “I performed fertility tests” with “fertility tests were performed”

line 933: delete extra “d” after “inserted”
line 966: fix typo in “complete”
line 1013: delete reference (Lidsky et al. 2013) and include in reference list
lines 1211-1212: suggest deleting “we again subtracted” (line 1211) and inserting “were subtracted” before the period (line 1212)
line 1531: replace “pores” with “pore”
line 1558: pluralize “Positions”
line 1567: replace “was” with “were”
lines 1602-1603: for clarity, perhaps rearrange to say “with (Mst27D+) or without (Mst27D-) endogenous Mst27D expression using the indicated antibodies…”
line 1662: replace “of” with “or”
lines 1686-1689: could the effect on Mst27D levels be due to instability of the Mst27D protein following knockdown of its binding partner Nup358?
lines 1723, 1725: replace “case of” with “the” (two instances)
line 1771: replace “CH” with “CT”
lines 1775-1776: confusingly worded: are the images in panels B-D all in a Mst27D- background or are some of them in Mst27D+?
lines 1809-1816: the descriptions of panels A and B are switched compared to the order of the images shown

All these problems are resolved in the revised version. We are embarrassed that evidently our final proof-reading of the original version has been so insufficient. Please apologize that these errors were present and be assured that we greatly appreciate the effort to point them out.
