# Supplementary material for: Nuclear elongation during spermiogenesis depends on physical linkage of nuclear pore complexes to bundled microtubules by Drosophila Mst27D
Source: PLoS Genet. 2023 Jul 10;19(7):e1010837. doi: 10.1371/journal.pgen.1010837 (PMC10359004; doi:10.1371/journal.pgen.1010837)
Supplement: Attachment — Submitted filename: comments.docx [file pgen.1010837.s024.docx]

Reviewer comments PGENETICS-D-23-00368

Across animals, spermatogenesis is characterized by packaging the highly condensed haploid paternal genome into a small sperm head. This involves dramatic changes not only in nuclear volume but also nuclear shape as exemplified by the formation of a needle like highly elongated nucleus in flies. This elongation is preceded by rearranging the distribution of nuclear pore complexes (NPC) along the nuclear envelope (NE) of the initially round spermatid nucleus in a way that NPCs cluster into one hemisphere of the nucleus that is surrounded by a MT rich environment (the “dense complex”) in the adjacent cytoplasm. The spatial proximity of NPCs and dense complex apparent in classical transmission electron microscopy (TEM) has suggested a functional link between the two structures but a molecular and functional proof for this hypothesis was missing.

The manuscript by Li et al resolves this question and offers a molecular explanation to a) how NPCs and dense complex are linked and b) how this connection is crucial for nuclear shape change during spermatogenesis. They find that the sperm specific EB-1 like protein Mst27D binds to microtubules (MT) and to the nucleoporin Nup358, a constituent of the NPC’s cytoplasmic filament, and thereby physically link the nucleus to MTs of the dense complex. They demonstrate the essential role for Mst27D since its absence affects nuclear elongation and shape, indicating that the NE is pulled via an NPC-MT linkage provided by Mst27D. They also show that Mst27D has MT bundling capacity, Consistent with this mst27D null mutants fail to form the prominent singular elongated MT bundle present in wild type spermatids. Overall these defects result in severely reduced fertility of male flies that lack Mst27D.

This work significantly improves our understanding of nuclear remodeling during spermatogenesis. It adds important mechanistic insight that is based on thorough biochemistry for the interaction network between Nup358, Mst27D and MTs and a detailed phenotypic characterization of *mst27D* null mutants. As such the manuscript should be published in principle, given that the authors can address the criticism listed below. In particular the authors should address the concern raised in point 7a) dealing with the interpretation of the nuclear elongation phenotype in *mst27D* mutants.

1) Figure 1:

The authors present the affinity pulldown with Mst27D::EGFP where they identify Nup358 and also its binding partner RanGAP. What about CG2233 ?? The authors should at least mention or discuss this apparently specific hit.

2) Figure 3: Interaction of Mst27D and Nup358

a) Figure3A: why does mcherry::Nup358 spread to MTs with FL-Mst27D but not with CT-Mst27D that actually binds Nup358 (Figure 2) and by itself also localizes to MTs around the nucleus (3A, middle panel) ?

b) How do Mst27D::GFP and cherry::Nup358 localize without co-transfecting the other protein? The authors should include this control.

c) the authors state that MT binding of Mst27D is mediated by its CH domain and homodimerization by its CC in the C terminus. Why? However in Figure 3A they show that Mst27D_C that has the CC but lacks the Calponin homology domain localizes to MTs. Why? The more clearcut answer to this question would be to transfect a Mst27D construct that just lacks the CC domain.

3) Figure 4

a) line 244: The authors write that their transgenes rescue mutants of *mst27D* or *nup358*. Which mutants were used (references !) ? What are the rescued phenotypes? Lethality or male sterility in the case of *mst27D* (where I suppose they use the mutant generated in this study)*?*

b) line 248: it would be helpful to relate the “S5”, “S6” staging as referred to in the results section (Figure 4, S4) to the initial description of sperm maturation, for example by explaining the “S” stages in Figure 1 or by text in the introduction.

c) expression of the Mst27D and Nup358 transgenes in spermatocytes: Could the authors show here that Mst27D localizes to the NE hemisphere that contains NPCs. Actually the authors do show this very nicely in Figure S6A where they present stills of a movie during NE polarization. I would suggest to move this particular display item (S6A, lower panel) into the main Figures (Fig 4) which would further illustrate their conclusion that Mst27D associates with NPCs (and is thus devoid of non NPC containing NE)

4. Dynamics of Mst27D

a) Figure S4:: the dendra::Mst27D should only spread within the NPC containing hemisphere. Can the authors comment on this?

b) the authors interpret their data on Dendra::Mst27D distribution after photoconversion as “intermittent episodes of transient association” with NPCs (line 263), because NPCs are believed to be immobile within the NE. Referring to this restraint, which was however discovered in somatic cells: how is eventually NPC distribution within the NE during spermatogenesis- which is very nicely demonstrated in Movie S1-achieved? Mobile NPCs, for example due to an altered lamina composition compared to somatic cells would not only allow NPC redistribution but also offer an alternative interpretation of Mst27D dynamics.

5) There is a major confusion

with the referring of results, main Figures and legends with respect to Figures 4, 5 and 6.

The text in the results section (starting from line 265) describes the strategy to knockdown by the degrade system. The respective data is presented in Figure 4B, C but the results section says Figure 5A and later 5B,C (line296). However Figure 5 presents results about nuclear elongation.

Later (from line 316) the text talks about results on nuclear elongation and NE shedding that are in fact presented in Figure 5A, yet the results section refers to Figure 6 (lines 319, 332).

6) Figure 5C and 5D

a) the authors present data obtained with peptide antibodies that they have raised to detect endogenous Mst27D. The antibody is specific, because the signal is gone in “Mst27D-“ tissues (Figure 5D). The legend to this Figure (line 1602) says “preparations of testes with or without endogenous Mst27D expression. This phrasing is ambiguous. The authors should state that it derives from the 0 mutant that they have generated which is presented in Figure 6.

b) With respect to the same figure (5D) I do not see which display item should support the statement made in line 345/346 that “a specific Mst27D signal was not observed before the onset of nuclear elongation”. I see only specific signal in all three panels (also in “early”), which is gone in Mst27D- tissue.

**7) Analysis of mst27D mutants**

a) The authors state that based on their analysis of mst27D mutants, the protein is required for nuclear elongation. This is well supported by the displayed stills of a movie from mst27D mutant spermatids expressing GFP::Nup358, compared to control spermatids (Figure 6E). However in the panels below (6F and 6G) nuclei in mutant spermatids appear also long, yet their shape is aberrant. The same holds true for Figure S9 or S12 B where, in later stages, nuclei seem to have well elongated despite the lack of Mst27D. Since the authors make a strong point in revealing the essential role of Mst27D for nuclear elongation they have to discuss or explain this discrepancy.

This discrepancy is also highlighted by the following sentence (line 564) where the authors discuss the independent roles of Spag4 and Mst27D: “ This preferential localization of Spag4-EGFP on the basal body increased further **during nuclear elongation in both controls and *mst27D* mutants** (S12B).”

b) In Figure S9 the authors rescue or do not rescue the nuclear elongation phenotype by expressing transgenes for versions of Mst27D. The figure should also contain a panel with just the mutant without any transgene to better assess the original phenotype

8) Figure 7

The authors should indicate the respective labels for the fluorescent proteins in panels B, C and D.

9) MT dynamics and bundling

while the name decolcemid is used in the text, the drug’s other name colcemid is used in the Figure legend (S10). To avoid confusion the authors should stick to one terminology.

10) Minor problems:

Line 146: the authors talk about Nup358 and its embedding in the NPC. They mention the NPCs outer ring, but non-experts won’t understand. A brief explanation of NPC architecture in the introduction would help, otherwise be less specific

Line 267: “ …that is assembled into **a** GFP-specific ubiquitin ligase”

Line 279: “For analysis we used testes dissected from larvae at the early pupal stages”. This is confusing, testes were from larvae or from pupae?

Line 409: … “nuclear elongat**ion** was limited…

Line 425: was also supported **by** the conclusion (for example)
